# Ocularity modulates the dominance of spatial over temporal influences in binocular rivalry

**DOI:** 10.1167/jov.26.10.9

**Published:** 2026-10-09

**Authors:** Lisa Beckmann, Thomas Schenk, Karin Ludwig

**Affiliations:** 1 Clinical Neuropsychology, Department of Psychology, Ludwig-Maximilians-Universität München, Munich, Germany

**Keywords:** bistable perception, flash suppression (FS), center-surround interactions, ocularity, spatial context, temporal context

## Abstract

The perception of a binocular rivalry (BR) stimulus can be influenced by other stimuli in temporal or spatial proximity. Here, we investigate how spatial (surround inhibition [SI]) and temporal context (flash suppression [FS]) interact. Previously, we modulated these influences by varying surround contrast in SI and timing parameters in FS. In the present study, we make use of the fact that the strengths of the two contexts can also be manipulated in the same way, namely by modulating ocularity. We varied FS strength by showing the prime to the same eye as the identical probe or to the other eye. Spatial influence was varied by presenting the surround to the same eye as the matching center, to the other eye, or to both. Temporal and spatial influences were mostly additive when biasing perception in the same direction. Some combinations were so strong that the dominant percept could be predicted almost perfectly. This may help in deliberately creating desired perceptual outcomes in future studies. Spatial context tended to dominate in conflicting situations. When both context types were presented interocularly (and presumably weaker), however, temporal context had the edge. This may reflect the unexpectedly strong interocular FS: the interocular constellation weakened FS much less than SI, suggesting differences in hierarchical processing levels. Thus, comparing interocular conditions may have been less informative than anticipated. Still, when the conflicting influences were both working intraocularly, spatial context dominated, consistent with previous findings.

## Introduction

Bistable perception has long intrigued scientists due to its implications for understanding visual processing, specifically how the brain selects a perceptual interpretation when the visual information from the environment is underdetermined. A method often used to examine bistable perception is binocular rivalry (BR). In this paradigm, two stimuli are presented to the two eyes at corresponding retinal locations. If the images are sufficiently different, perception alternates between those two images instead of merging them into a mixed version (Blake, 2022; Brascamp, Klink, & Levelt, 2015; Levelt, 1965).

BR has been shown to be influenced by the context in which it occurs (e.g., Brascamp, Knapen, Kanai, van Ee, & van den Berg, 2007; Kim & Kim, 2019; Lunghi & Alais, 2013; Mapperson & Lovegrove, 1991; Paffen, te Pas, Kanai, van der Smagt, & Verstraten, 2004; Wolfe, 1984). We know this from experimental research in which these influencing factors are studied – usually in isolation. Isolating individual influences is fundamental to experimental research, as it allows their effects to be characterized under controlled conditions. In natural vision, however, perceptual selection does not occur under the influence of a single stimulus property, modality or context factor. Rather, perception at any given moment is shaped by multiple concurrent and successive influences. Studying influences in isolation implicitly assumes that effects identified separately can subsequently be combined. This assumption may not hold if the relative strength of individual influences changes in the presence of another influence or if the influences interact. For example, one factor could strongly drive experimental results when studied in isolation but show only a weak influence when competing with another simultaneously acting factor. Examining multiple influences concurrently can therefore reveal their relative strength as well as dependencies between them that cannot be inferred from studying each influence separately. In an effort to open the field to investigating the interaction of different factors in influencing rivalry dynamics (or more generally, in shaping perception), we thus want to investigate the combination of two essential context factors: spatial and temporal context in the form of two well researched paradigms (see below and also see Beckmann, Schenk, & Ludwig, 2025b). We recognize that combining two experimentally controlled influences only captures a small fraction of the complexity of natural vision and that findings from this particular combination may not generalize to other influences. Nevertheless, we consider such combinations a step toward experimental designs that retain laboratory control while incorporating some of the complexity arising from multiple influences on perception.

Spatial context has been shown to exert a strong influence on rivalry dynamics. For example, presenting two bistable stimuli next to each other can lead to them alternating synchronously (e.g., Alais & Blake, 1999). If a bistable stimulus is presented next to a similar but unambiguous stimulus, the percept matched to the unambiguous stimulus will dominate perception (e.g., Dieter, Melnick, & Tadin, 2015). Alternatively, spatial context can also be provided in form of an annulus surrounding the bistable rivalry target, which, in the case of gratings (e.g., Beckmann, Schenk, & Ludwig, 2025a) or color (e.g., Paffen, Tadin, te Pas, Blake, & Verstraten, 2006), affects perception in a contrasting fashion, that is, the component of the bistable stimulus that differs clearly from the surround (e.g., the grating that is orthogonally oriented, of a different color, or of opposite motion direction) will be perceptually dominant. This is called the surround inhibition (SI) effect and is one of the most prominent examples of spatial context effects in bistable perception.

A widely researched example of temporal context effects is the flash suppression (FS) paradigm. In this paradigm, an unambiguous stimulus is presented, followed by a bistable stimulus consisting of the stimulus shown in the beginning and a rivaling one, sometimes the two are interrupted by a blank duration. It turns out that the blank duration between the two stimuli is critical. Short durations (including no blank) will lead to a transition from one percept to another (Beckmann et al., 2025a; Wolfe, 1984), whereas longer durations (>500 ms) will lead to a stabilization of the original percept (Brascamp et al., 2007).

The question of how these factors would interact shaped this current study and a previous one (Beckmann et al., 2025b). In our previous study (Beckmann et al., 2025b), the temporal context was provided in the framework of the FS paradigm, here, a BR probe (e.g., a clockwise [CW] oriented grating presented to the left eye and a counterclockwise [CCW] oriented grating presented to the right eye) was preceded by a prime, for example, a CW oriented grating presented to the left eye. Prime and probe could be separated by blank durations of different lengths (incl. 0 ms, i.e. no blank). As is common (Ikeda & Morotomi, 2002; Wolfe, 1984), the grating different from the prime (CCW in the example) tended to dominate perception. This effect was modulated by the blank duration (between prime and probe) but surprisingly not by the probe duration in the FS baseline. The spatial context was a surrounding annulus (around the central BR stimulus), which consisted of a grating that matched (i.e. iso-oriented) one of the central gratings. In the SI-only condition, we replicated typical effects that were observed in earlier SI experiments, that is, the stimulus different from the surround was mostly dominant and the strength of this inhibitory effect was modulated by the contrast of the surround (Beckmann et al., 2025b).

Importantly, when both paradigms were combined, their effects could be additive, with FS and SI boosting the same grating, or competitive, with FS and SI boosting different gratings. We found additivity as the occurrence of the boosted gratings was higher in combined additive trials compared with baseline trials (Beckmann et al., 2025b). This additivity indicated a stacking of effects in line with what we find for increasing sizes of the surround (Beckmann et al., 2025a; Xing & Heeger, 2001). In the competitive trials, the percepts corresponding to FS and SI preferences differed. Thus, the participants’ percepts provided valuable insight into which context – spatial or temporal – was more influential in resolving perceptual ambiguity. To obtain equally strong SI and FS parameters in these competitive trials, we matched conditions based on their baseline strengths and again found that, under equally strong conditions, spatial context remained the dominant influence on perception (Beckmann et al., 2025b).

Because SI and FS typically constitute distinct paradigms, the parameters adapted to modify strength differed substantially (e.g., surround contrast for SI vs. blank or probe duration for FS). Although we took care to match the strength of the individual contextual influences, it remains difficult to determine whether differences found in competition reflect the contextual influences themselves or differences in how influence strength was manipulated. Thus, in the current study, we decided to use a manipulation that works analogous for both paradigms: ocularity.

We use the term “ocularity” as an umbrella term to refer to the different constellations of the relationship of the influencing stimulus (prime in FS, or surrounding annulus in SI) with the influenced stimulus (BR probe; especially the matching grating, i.e. the grating that matches the prime or the surround). “Intraocular” describes constellations in which they are shown to the same eye (e.g., for FS when the prime and the matching grating [during the probe] are presented to the same eye). “Interocular” describes constellations in which they are shown to different eyes (e.g., for FS when the prime and the matching grating [during the probe] are presented to different eyes).

Previous work suggests that ocularity modulates both FS and SI. In FS, intraocular presentation generally produces stronger suppression than interocular presentation, although the magnitude of this difference varies across studies and, in some cases, was observed only under specific conditions (Brascamp et al., 2007; Ikeda, Ogata, & Morotomi, 2009; Ikeda & Morotomi, 2002). In FS, intraocular presentation is expected to produce stronger effects than interocular presentation because pattern- and eye-based effects align in the former but oppose each other in the latter. The strength of interocular FS suggests, however, that pattern-based effects contribute robustly to FS, even across eyes.

A similar pattern has been reported for SI. Studies using rivalry stimuli with surrounding contextual gratings have consistently found stronger contextual influences when surround and central target are presented intraocularly compared to interocularly (Mapperson & Lovegrove, 1991; Paffen et al., 2004). In addition, binocular surround conditions typically produce effects that are intermediate between intraocular and interocular presentation (Paffen et al., 2004) or of similar strength to intraocular presentation (Paffen, Alais, & Verstraten, 2005). These effects are commonly interpreted as reflecting lateral inhibition, whereby the surround suppresses neural responses to similarly oriented target gratings (Blakemore & Tobin, 1972). Together, these findings suggest ocularity systematically modulates both temporal and spatial contextual influences and therefore might provide a suitable common manipulation for comparing the two paradigms.

Manipulating ocularity in both spatial and temporal contexts is particularly interesting, as we would have the same expectation for the two effects: intraocular conditions should have a stronger influence than interocular conditions (for FS: Brascamp et al., 2007; for SI: e.g., Mapperson & Lovegrove, 1991; Paffen et al., 2004). In fact, in an early publication, Mapperson and Lovegrove (1991) wondered whether temporal and spatial effects were mechanically similar, in that they may share underlying adaptational processes. The direction of the influence would be decreasing in nature in both cases: in FS, the grating previously shown as a prime would be less dominant than the new grating; in SI, the surround-matching grating would be less dominant than the nonmatching grating.

As explained above, temporal context was manipulated by varying the ocularity of the FS paradigm. The strength of the spatial context was modulated by varying the ocularity of the annulus. For the FS baseline (trials without surround), we expected stronger suppression effects in the intraocular condition than in the interocular condition, that is, higher relative frequency of percepts in line with FS in the intraocular condition compared to the interocular condition.

For SI ocularity, we created three different levels: intraocular same (here, the surround is shown to the same eye as the matching grating (of the two central BR gratings), while the other eye sees a gray ring around the other central BR grating), binocular (the surrounding grating is presented to both eyes, matching one of the central gratings and being orthogonal to the other), and interocular same (the surround is shown to the eye that is presented with the nonmatching central grating, i.e. in one eye, the orientation of the surround grating is orthogonal to the orientation of grating in the center, while the other eye receives a central grating whose orientation is identical to that of the surround, but in this eye the central patch is surrounded only by a gray ring), see Figure 1. Based on previous results, we expect the interocular same condition to be the weakest. Generalizing findings from moving rivalry targets, the intraocular same condition is hypothesized to be stronger than the binocular condition (Paffen et al., 2004) or of similar strength (Paffen et al., 2005). Therefore, the SI ocularity effect is expected to increase in strength as follows: interocular same < binocular ≤ intraocular same. It should be noted that although BR is often characterized by sustained alternations between percepts, the current study focuses on onset-rivalry, that is, the initial selection of one percept following stimulus presentation. Therefore, we aim to extend this line of research by examining whether the pattern of results described above generalizes to static gratings and very short trials measuring onset rivalry (250 ms vs. 30 seconds).

**Figure 1.**
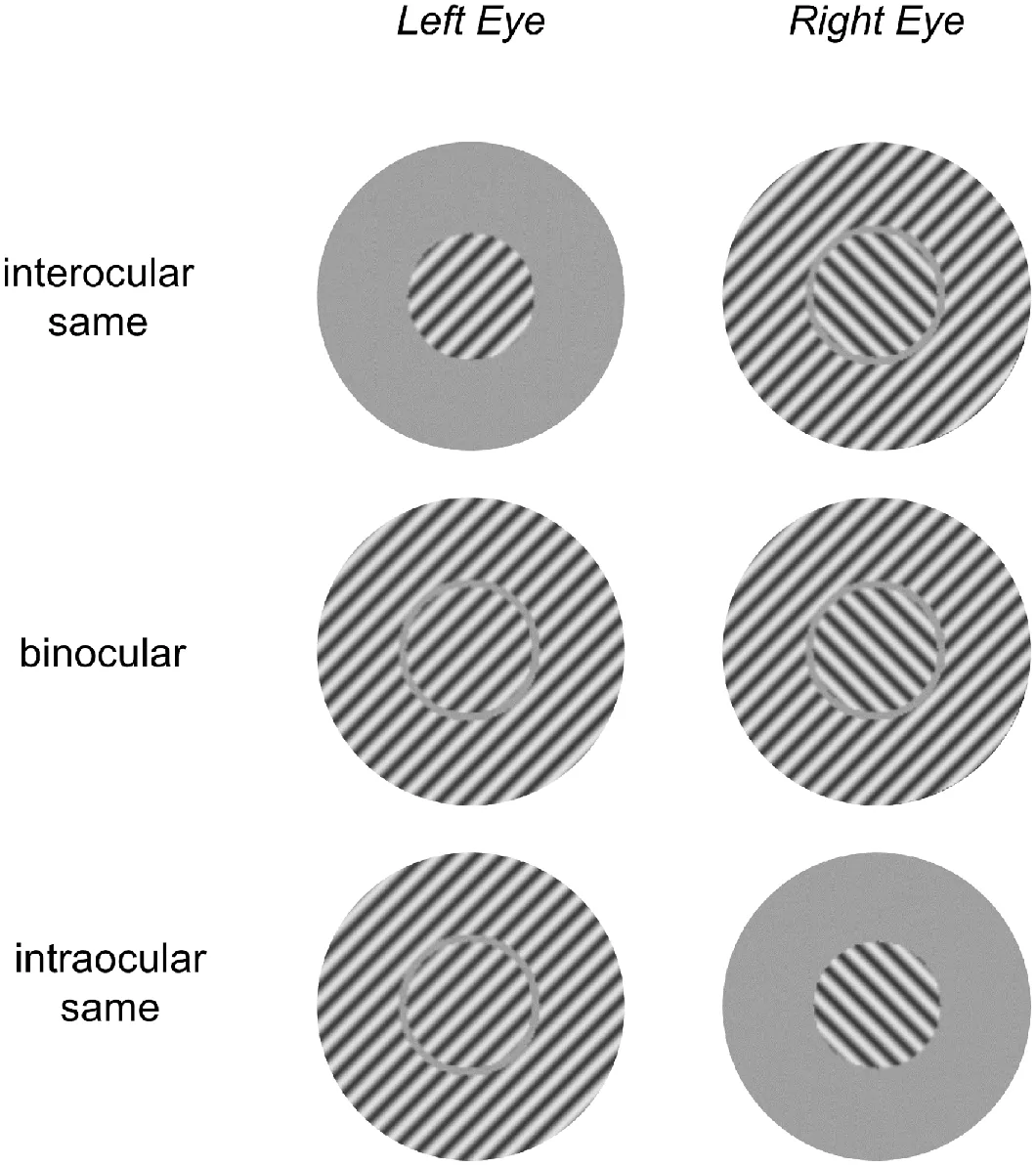
Example stimuli for the different SI conditions. *Note*: Example probe stimuli depicting the three SI conditions are shown. Participants reported their percept for the central stimulus. In the interocular same condition, the surround was presented only to one eye and orthogonal to the center. In the binocular condition, the surround was presented to both eyes with the same orientation and hence stable. In the intraocular condition, the surround was presented to one eye with the same orientation as the central grating.

The central aim of this study, however, is to look at the interaction between spatial and temporal context. Based on previous results (Beckmann et al., 2025b), we expect an additive effect between the two contexts, suggesting an interaction that follows the principle of many other visual phenomena. Furthermore, we want to test which context dominates perception, when put into competition. Based on previous results (Beckmann et al., 2025b), we expect the spatial context to be more important in competitive trials, allowing us to examine whether the previous results will also hold when relative strength of SI and FS are varied by manipulating ocularity conditions. In contrast to our previous experiments, in this experiment, conditions are not matched by baseline strength, but by how we manipulated the context; that is, we investigated how perception is affected when both SI and FS are intraocular (or both are interocular), and we also tried to ascertain whether spatial context prevails in these circumstances.

In many BR studies, observers are asked to choose among three perceptual options: perception of stimulus 1 (e.g., leftward-rotated grating), perception of stimulus 2 (e.g., rightward-rotated grating), or a mixture of both. In our experiments, observers were asked to first report the stimulus which was most clearly seen and then rate the clarity with which that stimulus was perceived. This procedure retains the forced-choice information about perceptual dominance, while providing a more graded measure of perceptual distinctness. Thus, the clarity rating can be understood as a quantitative extension of the traditional distinction between exclusive and mixed dominance (Blake, 2022; Dieter, Sy, & Blake, 2017; Yu & Blake, 1992), rather than a replacement for it. Higher clarity is assumed to correspond to more clearly resolved, less mixed perceptual experiences. So last, we expect participants to report greater clarity of their percepts when the two influencing factors are additive compared to when they are competing for perceptual awareness. Specifically, we will investigate whether this effect is due to percepts in additive trials being clearer than in the baseline, to competitive trials resulting in reduced clarity, or to a combination of both.

As a brief preview, our results demonstrate that examining these contextual influences in combination revealed properties that were not apparent from their individual effects alone. In particular, ocularity affected spatial and temporal context differently, whereas some combinations of both influences produced highly predictable perceptual outcomes despite the physical ambiguity of the rivalry stimulus – leading to almost perfect disambiguation.

## Methods

### Participants

A sample of 18 participants (nine female and nine male participants) took part, the sample size was chosen to match the previous study (Beckmann et al., 2025b). Ages ranged from 21 to 25 years, with an average age of 23.22 years (*SD* = 1.22 years). Most of the participants were recruited from the Ludwig-Maximilians-Universität München (LMU) and the Technische Universität München (TUM). Only right-handed participants who reported no history of psychological or neurological disorders were included in the study. All participants had normal or corrected-to-normal visual acuity. Compensation was provided as 10€ per hour or course credit. The study was approved by the local ethics board of the Department of Psychology of the LMU.

### Apparatus

All stimuli and the experiment were generated with Matlab R2018a, specifically using the Psychtoolbox version 3.0.16 toolbox (The MathWorks Inc., 2018) on an HP EliteDesk 800 G3 TWR computer. An AOC LCD monitor (27 in., 100 hertz [Hz] refresh rate) was used to present the stimuli. Stimuli shown on the right half of the screen reached the right eye and stimuli on the left half of the screen reached the left eye; this was achieved via a double-mirror stereoscope. The monitor was placed 56 cm from the participants’ eyes, including the distances within the mirror system (eyes to inner mirrors and inner to outer mirrors).

### Stimuli

Our stimuli were composed of a central sine-wave grating, and, in some conditions, a surround grating of equal spatial frequency and phase, see Figures 1 and 2, also refer to the Supplementary Figure S1. The center was the rivalry target and bistable when presented at the probe. The gratings were rotated 45 degrees CW (i.e. to the right) or CCW (i.e. to the left). The central grating was 0.83 degrees of visual angle (va) in diameter. Adding a surround increased the size to 1.5° va. All gratings had a Michaelson contrast of 90% and a spatial frequency of 8.43 cycles/° va. If a surround was present, a tiny gray gap spatially separated the central grating and the surround. This gap was introduced to ensure participants knew about which part of the stimulus to report their perception. The stimuli were surrounded by a dark gray fusion frame with black circles to reinforce binocular fusion. This fusion frame increased the size of the complete stimulus to 3.7° va. The mean luminance of all gratings and their respective position on screen was 100 cd/m^2^.

**Figure 2.**
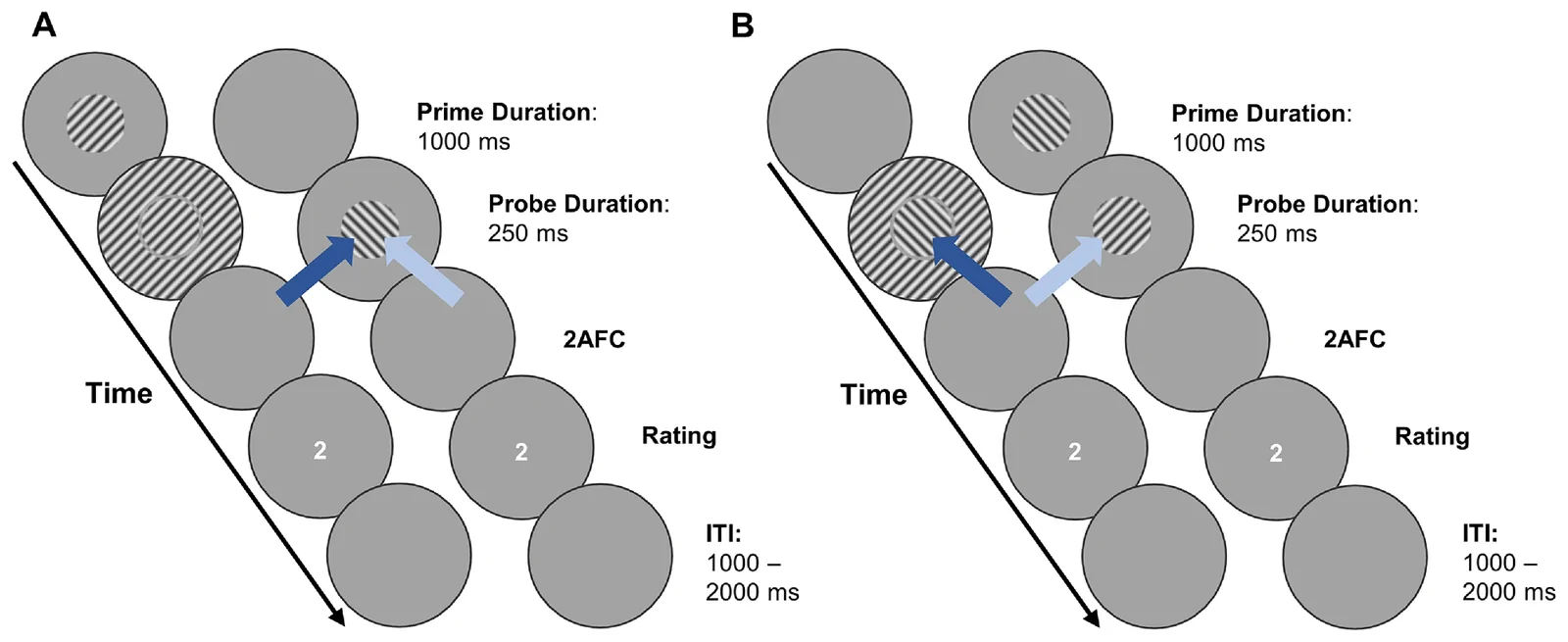
Example trials with an (**A**) additive and (**B**) competitive combination of contexts. *Note*: This figure shows two example trials with both spatial and temporal context present. The left circles represent what was presented to the left eye, and the right circles represent stimuli shown to the right eye. In the experiment, a circular fusion frame (not depicted here) surrounded these stimuli. In the 2AFC task, participants had to indicate the dominant percept during the probe. Afterward, participants recorded a perceptual clarity rating on a scale of 0 to 3. Each trial ended with an ITI randomly ranging from 1000 to 2000 ms. Dark blue arrows highlight percepts favored by SI, whereas light blue arrows highlight percepts favored by FS. (**A**) This trial is an example of an additive trial with intraocular FS and intraocular same SI. Both FS and SI favor the central CCW grating as it is orthogonal to the prime grating and the surround grating in the left eye. (**B**) This trial is an example of a competitive trial with interocular FS and interocular same SI. SI favors the CCW grating as it is orthogonal to the surround grating in the left eye, whereas FS favors the CW grating as it is orthogonal to the prime grating.

The surround was presented in three different ways (see Figure 1): the central grating could be completed by the surround in the same eye (intraocular same), in the other eye (interocular same), or in both eyes (binocular). In the FS baseline, no surround was presented. Instead, participants saw a gray ring with the same luminance as the central target. The stimuli and fusion frames were presented on a gray background with the average grating luminance, as recommended in the literature (Brascamp et al., 2007).

### Procedure

The experiment began with a simple spatial calibration procedure to find the optimal position for binocular fusion. Afterward, participants completed 12 practice trials to familiarize themselves with the task; these were not included in the analysis. The practice trials included examples from each relevant experimental condition. If participants reported many zero ratings for clarity in the practice, we asked them to check and possibly readjust the stimulus position and repeat the practice.

Each trial started with the prime stimulus, which was presented for 1000 ms, see Figure 2. The prime was always only shown to one eye, while the other eye saw an isoluminant gray patch. After the prime stimulus, the probe was shown for 250 ms. The probe was presented as two rivalrous sine gratings (CW- or CCW-oriented) in the center. In trials with a prime, one of the probe ratings was identical in orientation to the prime; it could be presented intraocularly (to the same eye) or interocularly (to the other eye). In trials with spatial context, a surrounding grating at either 10% or 90% Michaelson contrast was added. After the probe stimulus vanished, participants only saw gray patches, which served as their signal to indicate the orientation they had seen during the probe phase – left-arrow key for a CCW grating and right-arrow key for a CW grating. After the response, participants were asked to rate the clarity of the percept they had seen. Our clarity rating was an adaptation of the perceptual awareness scale (Ramsøy & Overgaard, 2004). Participants had been instructed for this before the start of the experiment and were asked to use the following assignment rules: 0 = “The percept was 50/50 and I might as well have reported the other”; 1 = “There was a small tendency for the reported direction, but the other was also relatively strong”; 2 = “It was relatively clearly this percept, but I also saw some of the other”; and 3 = “I only saw this grating.” The clarity rating was set by adjusting a number (starting at “2”) with the up- and down-arrow keys, then confirming it by pressing the space bar. The arrow-keys were operated with the right hand and the space bar with the left hand. Each trial ended with a randomized intertrial interval (ITI) ranging from 1000 to 2000 ms.

We included baseline conditions for each of the two paradigms, see Supplementary Figure S1. In the SI baseline condition, everything was the same as above; however, during the 1000 ms prime interval, no prime stimulus was presented (instead, a gray patch was shown to both eyes), and a surround was always present. In the FS baseline condition, no surrounds were presented. Otherwise, the procedure was the same as in the trials that combined spatial and temporal context. We had two relevant variables: (1) FS ocularity: intraocular (i.e. prime grating presented to the same eye during probe) and interocular (i.e. prime grating presented to the other eye during probe) and (2) SI ocularity: presentation of the surround (intraocular same, binocular, and interocular same). Additionally, we had three further control variables: (1) the grating orientation of the prime stimulus (CW and CCW), (2) the eye viewing the prime stimulus (left and right), and (3) surround grating: the grating type of the surrounding annulus (CW and CCW). Participants completed eight blocks of 44 trials each, combining 352 experimental trials, which took up to 60 minutes.

### Ensuring luminance uniformity

Monitors typically show variations of luminance across the screen. To account for this, we created two lookup tables, one for each monitor side, with gamma-corrected information for stimulus generation. This resulted in equally bright stimuli irrespective of position.

### Data pre-processing and analysis

To analyze the main outcome variable, that is, whether participants reported the predicted (1) or unpredicted (0) percept on each trial, we used generalized linear mixed-effects models (GLMMs) with binomial error structure and a logit link function. These models allowed us to assess binary responses while accounting for repeated observations within participants. For the analysis of clarity ratings, which were measured on an ordinal scale, we applied cumulative link mixed models (ordinal logistic regression) to appropriately handle the ordered categorical data.

All statistical models included a random intercept for each participant to model individual variability across trials. Fixed effects reflected the experimental factors relevant to each specific hypothesis, such as FS ocularity, SI ocularity, and trial type (additive, baseline, or competitive trials).

We evaluated model fit and contribution of fixed effects using likelihood ratio tests (LRTs) and comparisons of Akaike Information Criterion (AIC) values. McFadden's pseudo R^2^ statistic was reported as an indicator of effect size for LRTs involving nested models. Whereas pseudo R^2^ does not represent variance explained in the traditional sense, it reflects improved fit relative to a null model. McFadden (1973) noted that values between 0.2 and 0.4 suggest a strong model fit. To compare model fits, we reported ΔR^2^_McFadden_, that is, the difference between the R^2^_McFadden_ for the models being compared, and therefore the improvement in fit.

Where applicable, planned pairwise comparisons were conducted using estimated marginal means (EMMs), with Bonferroni adjustments applied to correct for multiple comparisons. In some analyses, Wald tests were used to evaluate whether model intercepts differed significantly from chance-level response probabilities (e.g., 50% in two-alternative forced choice [2AFC] queries).

For competitive trials, where spatial and temporal context favored conflicting percepts, responses were recoded according to predictions from the SI framework. This choice is arbitrary and we might as well have chosen prediction from the FS framework, as they are the inverse. In fact, for the figures, we break it down to show both sides for easier interpretation. Statistical models tested the independent and interactive effects of the contextual factors. To compare the relative contributions of FS and SI under comparable combinations, we also performed paired Bayesian *t*-tests.

All raw data and analysis scripts are publicly available https://osf.io/y65wd.

## Results

### Effects of ocularity in the baseline conditions

In the baseline conditions, we wanted to collect data to compare additive and competitive trials to test whether our implementation of the FS and SI paradigms yielded a general ocularity affect. To get a pure FS baseline, participants did not see any surround during the probe. In line with a general FS effect, we expected that the dominant percept during the probe would be the grating different from the one shown during the prime. To investigate this statistically, we fitted GLMMs with a binomial response (percept in line with FS [1] vs. not in line with FS [0]), including participant as a random intercept.

For this, we used a model that included only a random intercept for participants, with no fixed effects. A Wald test on the intercept showed that participants were significantly more likely to perceive the central grating that had not been shown during the prime, β = 2.52, *SE* = 0.29, z = 8.73, *p* < 0.001, 95% confidence interval (CI) = 1.95 to 3.08. This grating was dominant in 92.53% of trials. Thus, we could find the basic FS effect.

Following that, we aimed to show an effect of ocularity, where a stronger FS effect is observed when both the prime and (matching) probe are presented to the same eye (intraocular) compared to when presented to different eyes (interocular). To test this, we fitted another GLMM, including FS ocularity as a fixed effect and participant as a random effect. The interocular condition, hypothesized to produce the weakest effect, served as the reference. An LRT was used to compare this model with the simpler random-intercept model and indicated a significantly better fit, χ^2^(1) = 4.48, *p* = 0.034, R^2^_McFadden_ = 0.006, ΔAIC = 2.48. The intraocular condition showed a significantly higher estimated log-odds relative to the interocular reference (β = 0.43, *SE* = 0.20, z = 2.10, *p* = 0.036; see Figure 3A) and thus a stronger FS effect. These findings are consistent with prior results on FS ocularity (Ikeda et al., 2009).

**Figure 3.**
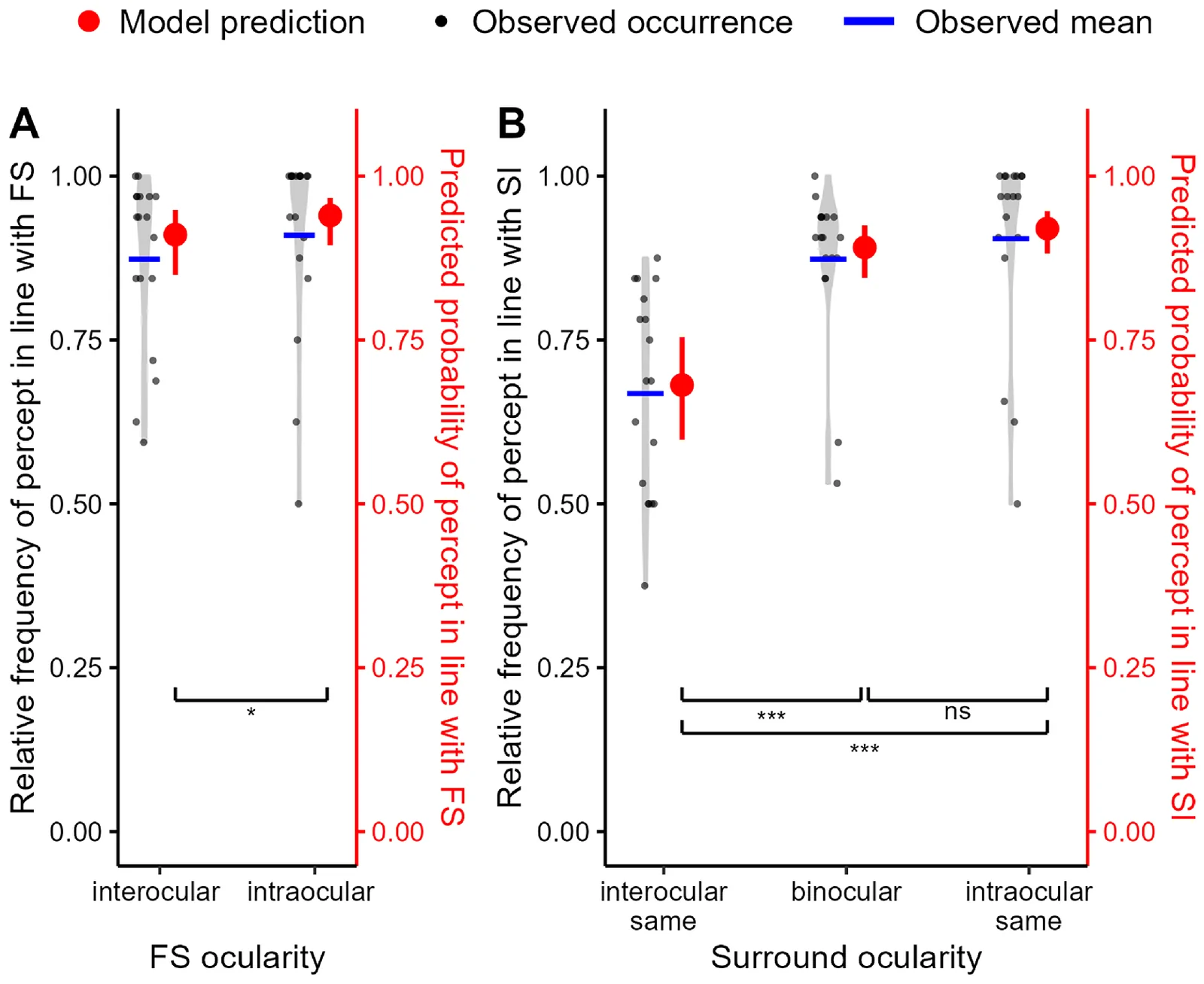
Baseline condition results for (**A**) FS and (**B**) SI. *Note*: (**A**) Relative frequency of the percept reported in line with flash suppression (FS) paradigm (black left y-axis) and corresponding model predictions (red, right y-axis) across varying ocularity conditions for the FS baseline condition (interocular and intraocular FS). Light-gray violin plots represent the distribution of individual data, with jittered black dots indicating the observed values per participant, and blue horizontal lines showing the observed condition means. Red points with error bars show model-predicted means and their 95% confidence intervals. Statistical significance is based on Bonferroni-corrected planned comparisons of model predictions; * = *p*_adj_ < 0.05, ** = *p*_adj_ < 0.01, *** = *p*_adj_ < 0.001. (B) Same as (**A**), but for the surround inhibition (SI) paradigm. Surround ocularity conditions are ordered from hypothesized weak (interocular same) through medium (binocular) to strong (intraocular same) in terms of hypothesized SI strength. All visual conventions from panel (**A**) apply.

In the SI baseline manipulation, participants should see the nonmatching central stimulus in most trials, that is, the stimulus orthogonal to the surround gratings. We again fitted two GLMMs – one with only the random effect of participant and another one that also included the fixed effect of SI ocularity. The response was binomial coded as “percept in line with SI” [1] versus “not in line with SI” [0]. The estimated intercept of the random-intercept model indicated that participants mostly perceived the nonmatching grating across different ocularity levels (83.17%), Wald test: β = 1.60, *SE* = 0.16, z = 10.02, *p* < 0.001, 95% CI = 1.29 to 1.91.

The model including surround ocularity provided a significantly better fit, LRT: χ^2^(2) = 129.90, *p* < 0.001, R^2^_McFadden_ = 0.082, ΔAIC = 125.90. Post hoc comparisons are plotted in Figure 3B: the SI effect was smallest in the interocular same condition. In both the binocular condition (β = 1.68, *SE* = 0.18, z = 9.58, adjusted *p* value (*p*_adj_) < 0.001) and the intraocular same condition (β = 1.34, *SE* = 0.16, z = 8.32, *p*_adj_ < 0.001) the SI effect was significantly larger. The comparison between the binocular and the intraocular same condition did not reveal any significant differences (β = 0.34, *SE* = 0.20, z = 1.74, *p*_adj_ = 0.247).

### Additivity of different ocularity conditions

When the two factors FS and SI influence perception in the same direction, we expect more percepts in line with FS and SI than in the baselines (only FS or only SI, respectively). We first analyzed the effect of spatial context (surround) on FS. To do this, we created a new factor called “additivity” to test for additive effects on the baseline. This factor had four levels: (1) FS baseline without any spatial context, (2) FS baseline with a spatial surround presented to the other eye than the matching central grating (SI: interocular same), (3) FS baseline with a binocular spatial surround, and (4) FS baseline with a spatial surround presented to the same eye as the matching grating (SI: intraocular same).

To test for the additivity effect, we fitted a series of GLMMs with binomial response (percept in line with FS [1] vs. not in line with FS [0]). Adding the additivity factor as a fixed effect improved the model fit significantly over the fit of the random-intercept model, LRT: χ^2^(3) = 21.38, *p* < 0.001, R^2^_McFadden_ = 0.013, ΔAIC = 15.38, implying a general additivity effect. This effect seems to be driven mostly by the addition of a binocular surround (β = 0.65, *SE* = 0.21, z = 3.15, *p*_adj_ = 0.005). Similarly, adding FS ocularity also improved the fit compared to the random-intercept model, LRT: χ^2^(1) = 7.48, *p* = 0.006, R^2^_McFadden_ = 0.005, ΔAIC = 5.48. Surprisingly, here, the supposedly “stronger” intraocular condition was in fact significantly weaker than the interocular condition, β = −0.74, *SE* = 0.16, z = −4.55, *p* < 0.001.

The best-fitting model was the full model, including FS ocularity and the additivity factor as fixed effects, as well as their interaction. The LRT compared this model to the model not including the interaction was significant, χ^2^(3) = 33.15, *p* < 0.001, ΔR^2^_McFadden_ = 0.021, ΔAIC = 27.15. Planned comparisons revealed that for interocular FS (see Figure 4, left panel), additivity could be found for the two stronger SI conditions (FS baseline + binocular SI: β = 1.57, *SE* = 0.36, z = 4.34, *p*_adj_ < 0.001; FS baseline + intraocular same SI: β = 1.57, *SE* = 0.36, z = 4.34, *p*_adj_ < 0.001), but not for the weakest SI condition (FS baseline + interocular same SI: β = 0.26, *SE* = 0.25, z = 1.05, *p*_adj_ = 0.886). Meanwhile, for intraocular FS (Figure 4, right panel), additivity could not be found. In one case (FS baseline + interocular same SI), participants even saw fewer percepts in line with FS (and SI) than in the FS baseline: β = −0.80, *SE* = 0.24, z = –3.39, *p*_adj_ = 0.002. This might just be the result of the intraocular FS baseline already reaching ceiling level or noise. However, Figure 4 suggests that interocular FS was indeed stronger than intraocular FS in additive trials. Thus, when FS was interocular, SI contributed to participants’ perception across all SI conditions, whereas for intraocular FS, SI did not have a further additive effect. This might again be due to already higher baseline values in the FS intraocular condition.

**Figure 4.**
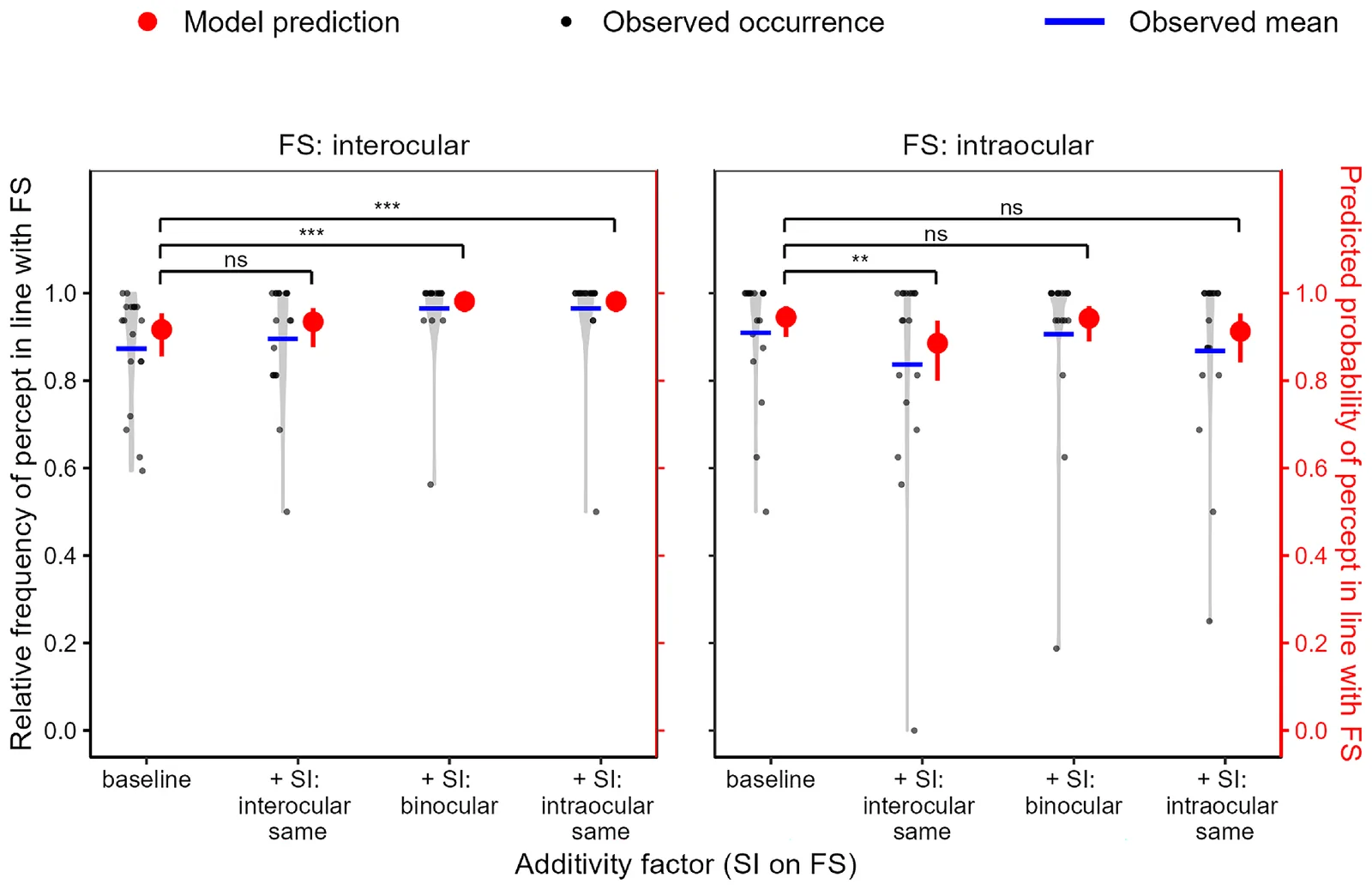
Additivity of spatial context (SI) on temporal context (FS). *Note*: Relative frequency of the percept reported in line with the flash suppression (FS) paradigm (black left y-axis) and corresponding model predictions (red, right y-axis) across different levels of the additivity factor. The x-axis is ordered from weakest to strongest, starting with the pure FS baseline to FS-baseline with the weaker SI condition (+ interocular same), then with a stronger SI condition (+binocular), and then with the strongest SI condition (+ intraocular same). Light-gray violin plots represent the distribution of individual data, with jittered black dots indicating the observed values per participant, and blue horizontal lines showing the observed condition means. Red points with error bars show model-predicted means and their 95% confidence intervals. Statistical significance is based on Bonferroni-corrected planned comparisons of model predictions; * = *p*_adj_ < 0.05, ** = *p*_adj_ < 0.01, *** = *p*_adj_ < 0.001.

When conducting the same analysis for the effect of temporal context (prime) on the SI, the effects got more complicated. Again, there were two fixed factors: the “surround ocularity” and the “additivity” factor. Here, the additivity factor had three levels: (1) SI baseline without anything being presented prior, (2) SI baseline with interocular FS, and (3) SI baseline with intraocular FS. Similarly to the previous analysis, the GLMM with the best fit was the full model, which included both fixed effects and the interaction. Including the interaction significantly improved the fit over a model including just the two fixed effects, LRT: χ^2^(4) = 23.78, *p* < 0.001, ΔR^2^_McFadden_ = 0.009, ΔAIC = 15.78.

Post hoc comparisons revealed significant additivity for the weak SI ocularity condition (interocular same) – SI baseline versus SI baseline + interocular FS ocularity: β = −1.70, *SE* = 0.23, z = −7.40, *p*_adj_ < 0.001; SI baseline versus SI baseline + intraocular FS ocularity: β = −1.10, *SE* = 0.20, z = −5.54, *p*_adj_ < 0.001. In the binocular ocularity condition, adding an interocular prime significantly increased the likelihood of predicted percepts (β = −1.54, *SE* = 0.36, z = −4.29, *p*_adj_ < 0.001), whereas an intraocular prime did not (*p*_adj_ = 0.384). A similar pattern arose for the intraocular same SI ocularity condition: adding an interocular FS prime significantly increased the occurrence of predicted percepts (β = −1.17, *SE* = 0.36, z = −3.22, *p*_adj_ = 0.004), whereas an intraocular prime did not (*p*_adj_ = 0.250). In summary, additivity could be demonstrated for all SI conditions, but especially in the weakest SI baseline condition (left panel in Figure 5). Interestingly, the results with the largest number of predicted percepts was found when SI was complemented by interocular FS (middle and right panel of Figure 5).

**Figure 5.**
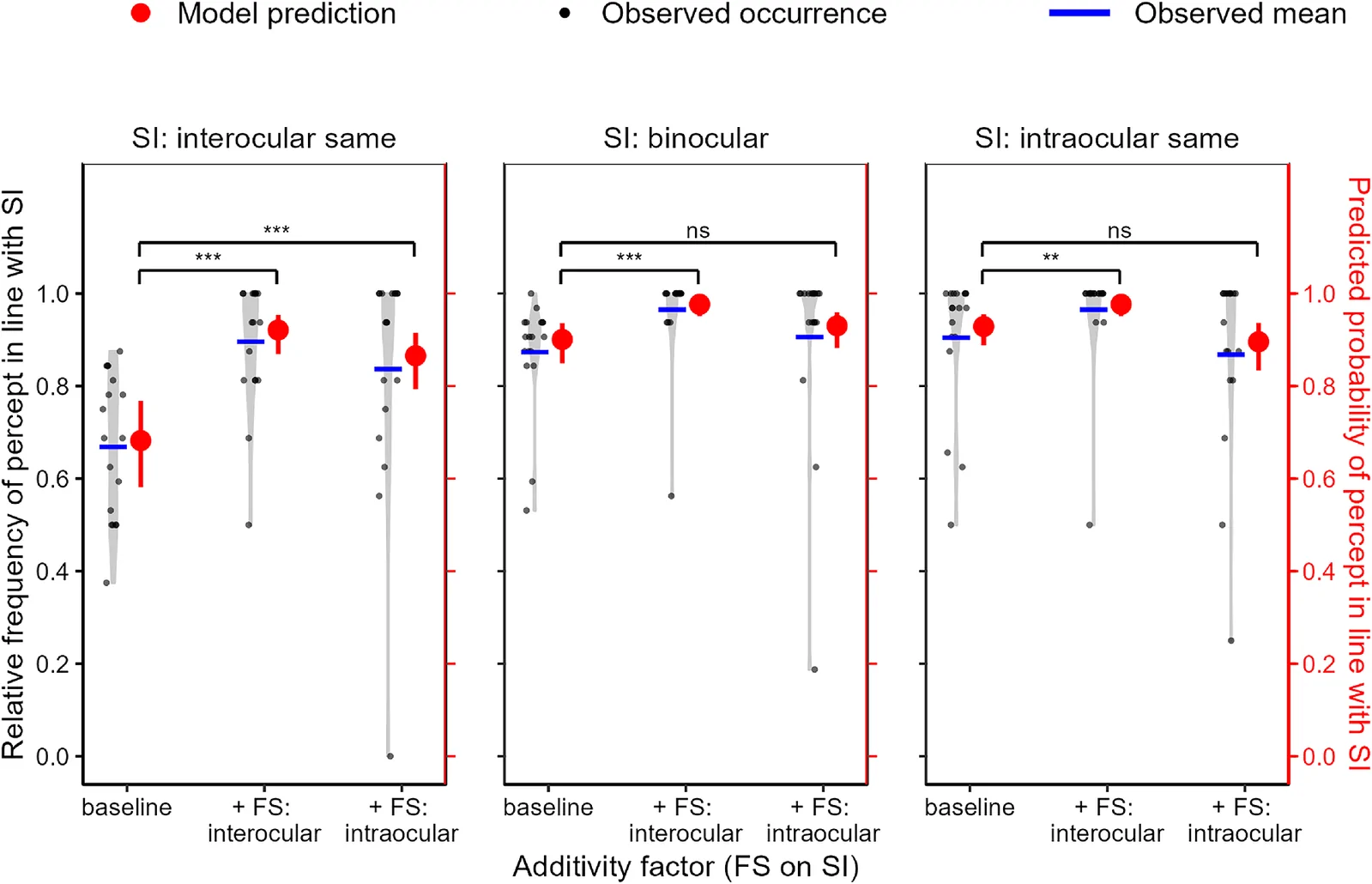
Additivity of temporal context (FS) on spatial context (SI). *Note*: Relative frequency of the percept reported in line with the surround inhibition (SI) paradigm (black left y-axis) and corresponding model predictions (red, right y-axis) across different levels of the additivity factor. The x-axis is ordered from weakest to strongest, starting with the pure SI baseline to the SI-baseline with the weaker FS condition (interocular) and then with the strongest FS condition (intraocular). Light-gray violin plots represent the distribution of individual data, with jittered black dots indicating the observed values per participant, and blue horizontal lines showing the observed condition means. Red points with error bars show model-predicted means and their 95% confidence intervals. Statistical significance is based on Bonferroni-corrected planned comparisons of model predictions; * = *p*_adj_ < 0.05, ** = *p*_adj_ < 0.01, *** = *p*_adj_ < 0.001.

### Binocular and intraocular same surround lead to spatial context dominance

Complementary to additive trials, there were also trials in which FS and SI influenced perception in different directions. For example, trials with a CCW-rotated surrounding grating (SI) and a CW-rotated prime grating (FS). Spatial context would boost the CW probe grating to dominate perception, whereas temporal context would favor the CCW grating. Investigating the actual percepts provides insight into how the relevant perceptual processes weight spatial versus temporal cues when selecting the perceptual output.

To investigate context dominance, we fitted a series of binomial GLMMs predicting whether the participant's response followed the SI-consistent percept [1] or not [0] (which equals: FS-consistent percept [0]/FS-inconsistent percept [1]). The models could include SI ocularity, FS ocularity, and their interaction term.

When comparing the models, we observed that including SI ocularity significantly improved the model fit compared to the random intercept model, χ^2^(2) = 143.35, *p* < 0.001, R^2^_McFadden_ = 0.064, ΔAIC = 139.35. This is driven by a weak interocular same condition (vs. binocular: β = 1.28, *SE* = 0.13, z = 9.70, *p*_adj_ < 0.001; vs. intraocular same: β = 1.38, *SE* = 0.13, z = 10.40, *p*_adj_ < 0.001). Similarly, adding FS ocularity also improved the fit over the random intercept model (χ^2^(1) = 26.25, *p* < 0.001, R^2^_McFadden_ = 0.012, ΔAIC = 24.25). In fact, predicted log-odds were higher for the intraocular FS condition compared to the interocular condition (β = 0.53, *SE* = 0.10, z = 5.10, *p* < 0.001). Given that the dependent variable is whether the response is in line with SI (the inverse of the response in line with FS), this means that – in combination with competing SI – FS was stronger in the interocular than in the intraocular condition. Adding FS ocularity to the spatial only model improved the fit (χ^2^(1) = 28.75, *p* < 0.001, ΔR^2^_McFadden_ = 0.013, ΔAIC = 26.75) and adding the spatial effect (SI ocularity) to the temporal only model (FS ocularity) also improved the fit, but much stronger (χ^2^(2) = 145.84, *p* < 0.001, ΔR^2^_McFadden_ = 0.065, ΔAIC = 141.84). Adding the spatial effect to the temporal model increased the model fit much stronger than the temporal effect on the spatial model as indicated by the higher ΔR^2^_McFadden_ and ΔAIC. This results from larger differences between condition in the spatial manipulation than in the temporal case already observed in the baseline. The best fit was provided by the model including also the interaction effect (χ^2^(2) = 14.67, *p* < 0.001, ΔR^2^_McFadden_ = 0.007, ΔAIC = 10.67).

Together with Figure 6, this significant interaction can be interpreted as follows: The spatial context was almost always the stronger context, except in the interocular same SI condition, which was the weakest spatial context condition. Here, FS exerted a stronger influence on perception than the spatial surround. Interestingly, the interocular FS condition (hypothesized to be weaker than the intraocular condition) was indeed stronger than the intraocular FS condition when in competition. Only here (in the interocular FS condition), was the difference of the competitive data from the FS baseline smaller than the difference of the competitive data from the SI baseline, that is, percepts were in line with FS, and spatial context could barely reduce this bias. The stronger effect of interocular FS compared to intraocular FS was particularly pronounced for the interocular same SI, but also present for the other two SI conditions.

We did not do baseline matching, like in our previous publication (Beckmann et al., 2025b), as the idea behind this experiment was to compare ocularity conditions. Thus, to decide which factor was stronger in competition, FS or SI, one must focus on the results in which both conditions were equally modulated, that is, the two interocular conditions (the left panel of Figure 6; both factors “weak”) and the two intraocular conditions (the right panel of Figure 6; both factors “strong”). In both cases, we calculated a two-sided Bayesian *t*-test against 0.5.

**Figure 6.**
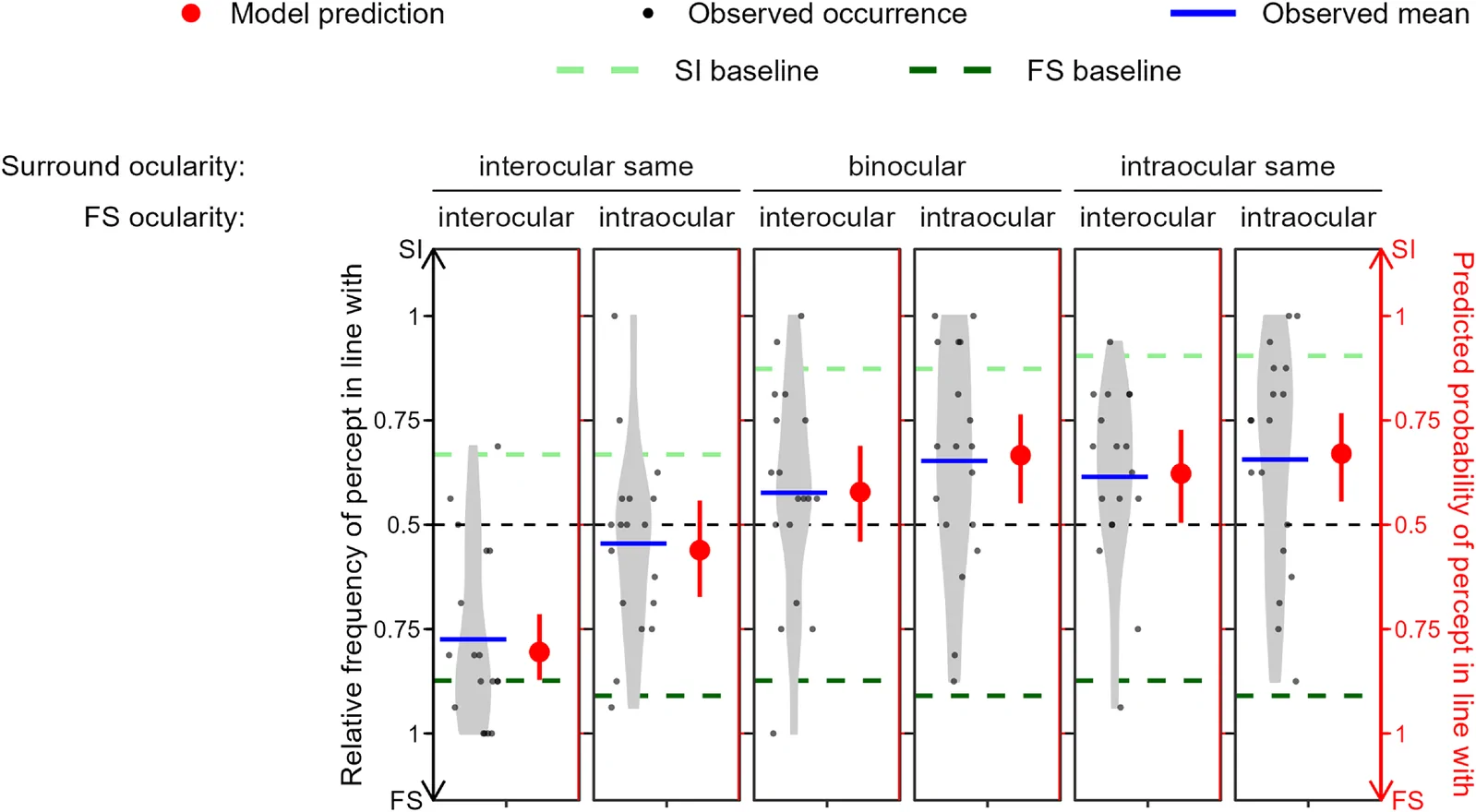
Results from competitive trials. *Note*: Relative frequency of the percept (black left y-axis) and corresponding model predictions (red, right y-axis) across different levels of the flash suppression ocularity and surround contrast. Light-gray violin plots represent the distribution of individual data, jittered black dots indicate the observed values per participant, and blue horizontal lines show the observed condition means. Red points with error bars show model-predicted means and their 95% confidence intervals. As in Figure 5, the 0.5 on the y-axis means that SI and FS had the same influence on perception (visualized by a black dashed line). Values above this line indicate perceptual effects consistent with SI dominance, whereas values below this line reflect FS dominance. For reference, the average SI baseline values were plotted as light green dashed lines and average FS baseline values as dark green dashed lines. Only in the two leftmost panels (SI: interocular same) is the perception mostly in line with FS. In addition, only in the leftmost panel (SI: interocular same, FS: interocular) is the difference from the FS baseline smaller than the difference from the SI baseline. The spatial dominance in all other conditions confirms previous results.

In the first case (interocular), FS, the temporal factor exerted a stronger dominance with decisive evidence in favor of this effect (see Figure 6, leftmost panel, BF_10_ = 597.24). The fact that ocularity modulated FS very weakly becomes relevant in this context; in fact, the interocular FS effect (leftmost panel) was even stronger than the intraocular FS effect (second panel from the left, Figure 6). In SI, however, the interocular condition was quite weak indeed, both in competition as well as in the baseline (see Figure 3 and light green dashed line in Figure 6). In fact, in the baseline conditions (see Figure 3), the interocular FS condition was considerably stronger than the interocular (same) SI condition (BF_10_ = 963.37). When comparing competitive trials from this condition, it is important to account for this difference in strength.

In the second case (intraocular), SI, the spatial factor, was stronger than FS (see Figure 6, rightmost panel, BF_10_ = 2.63), with anecdotal evidence for this effect. In this condition, however, the spatial and temporal factors were on more equal footing: comparing the respective intraocular baseline conditions revealed very similar strengths (FS = 91.07% and SI = 91.99% model estimates, with moderate evidence for equality, BF_01_ = 4.08). In this, thus fairer comparison, SI had a stronger influence on perception.

To sum up our results, competitive trials with ocularity manipulations paint a more complex picture compared to when contrast was manipulated (Beckmann et al., 2025b). Spatial context was always stronger except in its weakest condition (interocular same). Consequently, when both effects worked intraocularly (FS: intraocular and SI: intraocular same), there was a preference for spatial context. The only clear preference for temporal cues could be found in the interocular same SI condition in combination with the interocular FS condition, which was stronger than anticipated, but also differed in baseline strength. Interestingly, no clear preference could be found in the supposed stronger FS condition (intraocular) in combination with the interocular same SI condition (see Figure 6, second panel from the left), even though the spatial context was considerably weaker in the baseline.

When looking at the distance between the data in the competitive condition (see the blue lines, Figure 6) and the respective baselines (SI: light green and FS: dark green dashed lines), the picture becomes even clearer: except in the interocular FS/interocular SI condition, the data are closer to the SI baseline than to the FS baseline.

Concerning the strengths of intra- versus interocular FS, Figure 6 reflects what we have already found in the FS baseline: pattern-based effects are so strong that adding eye-based effects does not substantially strengthen the effect consistently, in fact, the interocular FS condition seemed more resistant against influences of the spatial context compared to the intraocular FS condition.

### Clarity ratings: Additive versus competitive trials

Previously, we demonstrated how additive and competitive conditions affect the dominant percept. Next, we investigated how the trial type (competitive, SI baseline, FS baseline, and additive) affected the clarity of these percepts. To achieve this, we conducted two analyses. In the first, we compared clarity ratings in competitive trials, the SI baseline, and additive trials, and examined how these ratings were affected by surround ocularity. To capture the clarity of the relevant percept, we used the following transformation: when the first response (which grating was dominant?) was in line with SI, the rating remained the same. If the response was in line with FS, we applied sign reversal by multiplying it by –1 to obtain a negative value. This transformation is particularly important as clarity in competitive trials could reflect the clarity of the FS percept, particularly in weak SI conditions. Because there is no FS ocularity manipulation in the SI baseline and no SI ocularity manipulation in the FS baseline, two separate analyses were conducted. In the first part, we aimed to determine if competitive trials exhibit lower transformed clarity compared to the SI baseline and whether the additive trials would have higher transformed clarity than the SI baseline trials.

We fitted a series of cumulative link mixed models (CLMMs) predicting the clarity rating with a random intercept for participants. Beginning with a model including solely the trial type as a fixed effect, we found this model to improve the model fit compared to the random-intercept model, LRT: χ^2^(2) = 945.10, *p* < 0.001, R^2^_McFadden_ = 0.057, ΔAIC = 941.10. This effect of trial type is driven by both a lowered clarity for competitive trials (β = −1.19, *SE* = 0.06, z = −18.75, *p*_adj_ < 0.001) and an increased clarity in additive trials (β = −0.84, *SE* = 0.06, z = −12.59, *p*_adj_ < 0.001), see Figure 7A. Comparing a model with SI ocularity also improved the fit compared to the random-intercept model: LRT: χ^2^(2) = 219.30, *p* < 0.001, R^2^_McFadden_ = 0.013, ΔAIC = 215.30. Here, we found that interocular same produced less clarity compared to the binocular condition (β = −0.89, *SE* = 0.06, z = −13.81, *p*_adj_ < 0.001), which in turn produced a lower clarity than the intraocular same condition (β = −0.16, *SE* = 0.06, z = −2.52, *p*_adj_ = 0.024).

**Figure 7.**
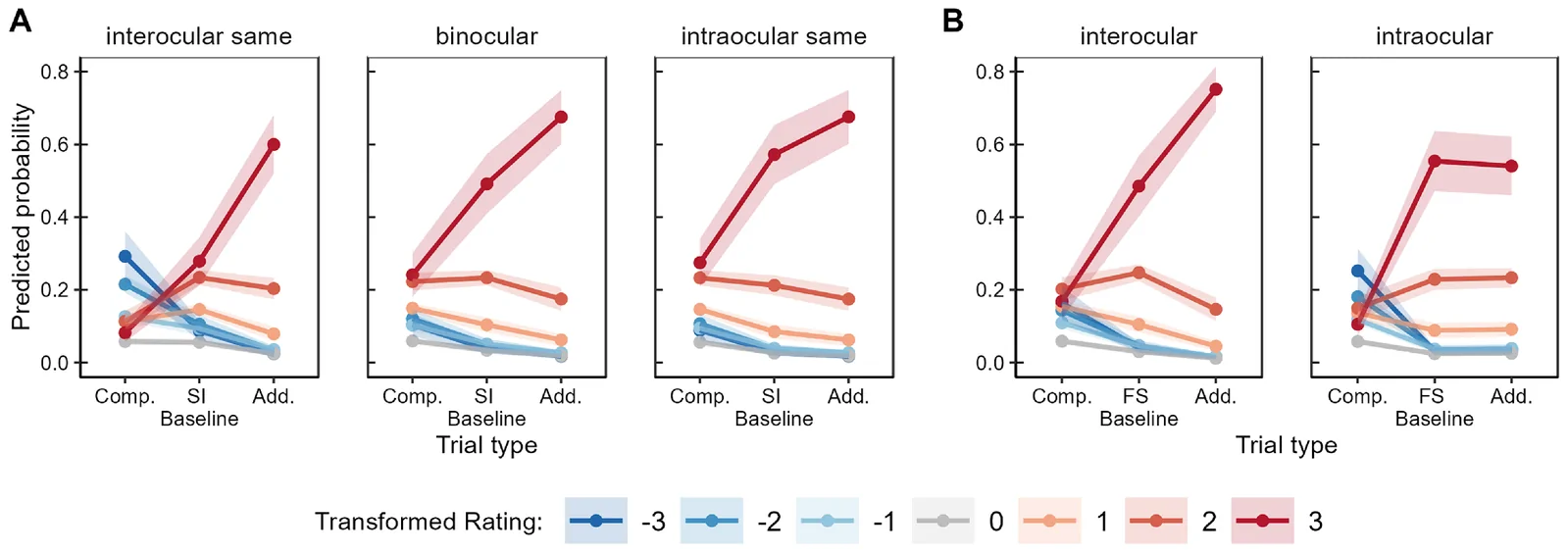
Model prediction for the transformed clarity ratings. *Note*: Predicted probabilities of transformed clarity ratings (–3 to 3) from a Cumulative Link Mixed Model (CLMM), as a function of trial type (x-axis) at different (**A**) surround ocularity, and (**B**) FS ocularity conditions. Positive ratings (warm colored lines) represent ratings after responses in line with the respective influence (A: SI, B: FS), negative ratings (cold colored lines) represent ratings after responses not in line with this respective influence, the higher the absolute value of this rating, the higher the expressed clarity. Dots represent predicted probabilities for each rating level, with shaded ribbons indicating 95% confidence intervals (lines between the dots were only introduced for clearer presentation). Higher values on the y-axis indicate greater model-predicted likelihood of a given clarity rating. The equivalent plot with the participants’ data (instead of model estimates) can be found in the Supplemental Material S.3, Supplemental Figure S7.

The model providing the best fit was the full model including both fixed effects as well as their interaction, LRT comparison to the model including only SI ocularity and trial type but no interaction: χ^2^(4) = 58.92, *p* < 0.001, ΔR^2^_McFadden_ = 0.004, ΔAIC = 50.92, see Figure 7A. It seems like the contribution of the interaction is relatively minimal, as indicated by a relatively low ΔR^2^_McFadden_.

In the mirror analysis, we compared competitive trials with the FS baseline and the FS baseline with additive trials per FS ocularity level and found a similar pattern of effects. Similarly to the previous clarity analysis, we first compared a model including trial type with the random-intercept model and found a significantly better fit, LRT: χ^2^(2) = 1400.17, *p* < 0.001, R^2^_McFadden_ = 0.095, ΔAIC = 1396.17. We found that the competitive trials had a lower clarity compared to the baseline trials (β = −1.93, *SE* = 0.07, z = −25.91, *p*_adj_ < 0.001), which in turn had a lower clarity than the additive trials (β = −0.51, *SE* = 0.07, z = -6.89, *p*_adj_ < 0.001). A model including FS ocularity also provided a better fit than the random-intercept model, LRT: χ^2^(1) = 46.28, *p* < 0.001, R^2^_McFadden_ = 0.003, ΔAIC = 44.28. Interestingly, this effect was driven by a lower clarity in the “stronger” intraocular condition, compared to the “weaker” interocular condition (β = −0.37, *SE* = 0.05, z = −6.79, *p* < 0.001).

In the best fitting model including the interaction of trial type and FS ocularity (compared to the model without the interaction: χ^2^(2) = 68.91, *p* < 0.001, ΔR^2^_McFadden_ = 0.005, ΔAIC = 64.91), competitive trials showed a lower clarity compared to the baseline in both the weak interocular FS condition (β = −1.54, *SE* = 0.10, z = −15.18, *p*_adj_ < 0.001) as well as the strong intraocular FS condition (β = −2.36, *SE* = 0.10, z = −22.69, *p*_adj_ < 0.001). The comparison between FS baseline and additive trials only became significant in the interocular FS ocularity condition (β = −1.17, *SE* = 0.11, z = −10.62, *p*_adj_ < 0.001), see Figure 7B. Overall, competitive constellations of spatial and temporal context indeed led to lower clarity and additive constellations mostly led to higher clarity.

### Supplemental experiment: Replication of main findings in previous dataset

To evaluate whether the present findings generalize beyond the current experiment, we conducted supplementary analyses on a separate dataset collected using a related paradigm that combined FS and SI through manipulations of ocularity and surround contrast, respectively. For reasons of brevity, these analyses are reported only briefly here; see the Supplementary Materials S2 for a more detailed analysis.

The results were broadly consistent with those reported above. First, robust FS was observed in the baseline condition, with stronger effect for intraocular than interocular presentation. Second, the general additivity of spatial and temporal context effects was further confirmed. At least in the weaker conditions, respectively, the added second factor (FS) led to increased influence on perception. Additionally, for the high contrast condition, the comparison between the SI baseline and the SI baseline with an interocularly presented FS prime demonstrated additivity. Therefore, whereas additivity is most prominent in weak conditions, it is not restricted to them. In several conditions, perceptual dominance exceeded 95%, indicating particularly strong additive effects. Third, when temporal and spatial context favored different percepts, spatial context was again shown to be the stronger influencing factor. Using a pair of baseline conditions that showed the greatest similarity in baseline performance as a reference, spatial context exerted the stronger influence under competitive conditions. In the case of low contrast surrounds, however, FS mostly determined perception (in both FS conditions, intra- and interocular). Together, these findings suggest that the effects reported in the present experiment are not restricted to a specific implementation of SI strength manipulations but generalize across different experimental manipulations of spatial context.

## Discussion

Here, we aimed to investigate the influence of ocularity on both FS and SI, as well as the combination of both context types. As expected, we found the interocular FS condition to produce a smaller FS effect than the intraocular FS condition in the FS baseline, albeit with a relatively small difference. In the SI baseline condition, we found that the intraocular same condition and the binocular condition yielded stronger SI effects compared to the interocular same condition. Thus, it is important to note that the outcome of our manipulation of ocularity differed partially from what we had intended. Whereas the intraocular conditions for SI and FS produced comparable effects in their respective baseline measures, the interocular manipulation did not behave as expected. In particular, FS exerted a much stronger influence on perceptual outcomes than SI in the corresponding interocular baseline condition. This asymmetry, although interesting, complicates direct comparisons in the competitive trials and should be kept in mind when interpreting the following results.

To characterize additivity of the temporal and spatial factor, two analyses were carried out. In the first analysis, we used an FS-only condition as a baseline and examined how performance was affected by spatial context (see Figure 4). The additivity effect was modulated by ocularity: specifically, we found pronounced additivity only when (strong) SI-context was added to the interocular condition of the FS paradigm, not in the intraocular FS condition. This might be explained by already higher values in the intraocular FS baseline. In the second analysis, we used an SI-only condition as our baseline and examined how performance was affected by adding the temporal context (FS). In this condition, we found additivity which was again modulated by ocularity. Specifically, we found that when SI was weak, temporal context provided an additive effect, irrespective of FS ocularity. Somewhat surprisingly, in the strong SI conditions (i.e. intraocular same and binocular), it was the weaker interocular FS condition that produced additivity, whereas the stronger intraocular FS condition did not produce a significant additive effect, a pattern mirrored in the supplemental data. Taken together, we found strong interactions between additivity and ocularity. It is noteworthy that sizable additivity was found despite the fact that the baseline dominance for the predicted percept was already quite high.

In the competitive trials, we again found that the level of the spatial context, that is, SI ocularity, was the determining factor for which percept dominated perception. In contrast, the manipulation of the temporal context (FS ocularity) played a minor role. A stronger effect for temporal context was only found in one condition combination, FS: interocular, SI: interocular same, that is, when spatial context was weak and FS was also in its supposedly weaker, but – in fact – stronger, condition. When both were presented intraocularly (FS: intraocular and SI: intraocular same), spatial context was the stronger influence. Investigating the clarity ratings, we found a significant reduction in clarity for competitive trials, as well as an increase in clarity for additive trials compared to their respective baseline conditions in both FS and SI. Before turning to the interpretation of the ocularity findings, it is worth noting that the overall pattern of results was consistent with the supplementary analyses reported in Supplemental Material S.2. Despite using surround contrast rather than ocularity to manipulate SI strength, the supplementary experiment likewise demonstrated additive effects between temporal and spatial context, a predominance of spatial context in competitive conditions, and systematic changes in perceptual clarity. Thus, the conclusions drawn (except those pertaining to SI ocularity) are supported by two independent datasets that used different manipulations of SI strength.

### Ocularity affects SI more strongly than FS

Although ocularity could be manipulated analogously in both paradigms, the present findings suggest that eye-of-origin information does not contribute equally to temporal and spatial contextual influences.

So far, only a few studies have compared inter- and intraocular prime-probe presentation in FS. Ikeda and Morotomi (2002) reported very strong FS in both intra- and interocular conditions using chromatic stimuli, demonstrating robust interocular transfer of the FS effect. Ikeda et al. (2009) also examined intra- versus interocular FS using oriented gratings and found stronger effects for intraocular presentation, but also relevant interocular effects. Meanwhile, Brascamp et al. (2007) found complex results for ocularity interacting with stimulus contrast and prime duration. For the stimulus conditions closest to ours (stimulus contrast 100%, 1000 ms prime, and 71 ms blank), they found strong FS in the intraocular condition but not in the interocular condition. Thus, across studies, FS was consistently observed in the intraocular condition. More mixed results were obtained for FS in the interocular condition, for example, Brascamp et al. (2007) failed to find interocular FS under some conditions (i.e. those conditions that were most similar to those used in our experiment); Ikeda et al. (2009) found that FS was weaker for interocular than intraocular conditions; Ikeda and Morotomi (2002) reported similar FS effects for both inter- and intraocular conditions in one condition.

We observed strong FS for the interocular condition, which was only slightly reduced compared to the intraocular condition in the baseline trials. When we take the effects of ocularity on additivity and competition between contexts into account the relationship between ocularity and dominance becomes quite complex. Additivity for spatial and temporal context is only found when FS is presented interocularly. In competitive trials, the interocular FS context competed more strongly with SI than the intraocular context. This pattern may partly reflect interactions between processes operating at different levels of the visual hierarchy. Nevertheless, the overall picture is that the influence of FS context remains strong under interocular conditions. This suggests that ocularity has less of an impact on FS influence than SI influence. Such a pattern of findings is compatible with the assumption that FS is mainly driven by pattern-based influences rather than eye-based influences. Switching from the interocular condition, in which only pattern-based influences support the effect to the intraocular condition in which both pattern- and eye-based influences act in the same direction, produced only modest changes in perceptual outcome. The persistence of substantial interocular FS therefore suggests a strong contribution of higher-level stimulus representations. This might point to an interesting difference between the influence of FS and SI contexts.

Previous studies on SI have shown that it is consistently stronger when the central and surrounding gratings are presented to the same eye than when they are presented to different eyes. This pattern has been observed for both static (Mapperson & Lovegrove, 1991) and moving gratings (Paffen et al., 2004). In the latter study, where the rivalry target was a moving grating drifting either leftward or rightward, Paffen et al. (2004) found stronger SI effects in the intraocular same condition than in the binocular same condition, which in turn produced stronger SI effects than the interocular same condition. In a subsequent study, Paffen et al. (2005) used a moving grating versus a static concentric grating as rivalry targets, yielding a somewhat different pattern. Although they did not explicitly compare binocular same, monocular same (equivalent to our intraocular same), and interocular same conditions, their published figures suggest comparable effect sizes for the binocular and monocular same conditions. In contrast, the interocular same condition appeared noticeable weaker, producing roughly half the effect. Despite methodological differences between their study and ours, including the use of moving stimuli, predominance measures, and substantially longer viewing durations, we observed a broadly similar pattern. We found no significant difference between intraocular same and binocular same conditions, although the former tended to be numerically larger. Both conditions, however, produced stronger SI effects than the interocular same condition.

This convergence suggests that the critical determinant of SI is whether center and surround signals engage the same monocular channel. Such a pattern is consistent with accounts emphasizing lateral inhibition between neurons tuned to similar stimulus features (Blakemore & Tobin, 1972). If center and surround information are presented to different eyes, the effectiveness of the inhibitory interactions appears to be substantially reduced. Consequently, SI seems to depend more strongly on processes that preserve information about the ocular relationship between contextual and target stimuli. A possible computational interpretation of the similarity between the “intraocular same” and the “binocular” condition, comes from interocular normalization models of binocular processing. Using stimuli not dissimilar to our surround, Moradi and Heeger (2009) showed that responses in the primary visual cortex can be explained by normalization between the two eyes, with binocular and monocular stimulation producing very similar activity once stimulus contrast is sufficiently high (as was the case in our experiment). The similarity between binocular and intraocular SI thus may reflect comparable processing at an early binocular stage, whereas the weaker interocular-same condition suggests that eye-specific influences remain important before binocular signals are fully integrated.

Taken together, our results indicate that ocularity modulates SI more strongly than FS. Although FS showed relatively similar strengths in inter- and intraocular configurations, SI consistently depended on whether center and surround were presented to the same eye. Thus, although ocularity provides a useful common experimental manipulation, the present findings indicate that eye-of-origin information constrains temporal and spatial contextual influences in different ways.

### Additivity in temporal and spatial context dynamics

In the additive conditions of the main and the supplemental experiment, the overall pattern largely replicated our previous findings (Beckmann et al., 2025b): when temporal and spatial context favored the same percept, the combined influence generally exceeded that observed in the respective baseline conditions. This finding further supports the notion that temporal and spatial contextual influences can accumulate to shape perceptual selection.

Notably, we also found additivity despite already high baseline levels. In several conditions, combining temporal and spatial contextual manipulations resulted in perceptual dominance rates exceeding 95% with the highest result being 96.50% in the interocular FS and intraocular SI condition. This indicates that contextual manipulations can produce highly reliable perceptual outcomes in participants while preserving the physical ambiguity of the rivalry stimulus. This observation may have practical implications for research on bistable perception. Disambiguation is often achieved by physically altering a stimulus (Brouwer & van Ee, 2007; Grossmann & Dobbins, 2003; Hasson, Hendler, Bashat, & Malach, 2001; Weilnhammer, Ludwig, Hesselmann, & Sterzer, 2013) or presenting unambiguous replay conditions (e.g., Blake & Fox, 1974; Brascamp, Sterzer, Blake, & Knapen, 2018; Knapen, Brascamp, Pearson, van Ee, & Blake, 2011). However, such manipulations do not necessarily reproduce the subjective experience associated with genuinely ambiguous stimuli (O'Shea & Corballis, 2004). In contrast, the present paradigm demonstrates that combining temporal and spatial contextual influences can strongly bias perception while leaving the physical properties of the rivalry stimulus unchanged. This approach may therefore provide a useful alternative when researchers wish to increase perceptual predictability without abandoning bistability altogether.

Furthermore, we observed the following interesting pattern of results. When spatial context was strong (main experiment: binocular, and intraocular; supplemental experiment: 90% contrast), only interocular FS produced additivity. This might be explained by considering what happens at the monocular level. Let us assume that SI mostly works through lateral inhibition of the matching grating (both in binocular same and intraocular same conditions) and that FS works through adaptation to the matching (prime) grating and eye, but that pattern-based effects (i.e. adapting to the grating) are more important than eye-based effects. When looking at SI, let us focus on the neural monocular representation of the matching grating. Let us further assume that a significant portion of SI happens at this level (this is confirmed by our big differences between intra- and interocular SI). In case of preceding intraocular FS, this representation has already been weakened by previous adaptation (at a monocular level). Monocularly, SI then acts on an already weakened grating, making these SI processes possibly less strong. In case of interocular FS, this representation has not been weakened yet (because this weakening happens at later binocular stages through pattern-based effects). This leaves a stronger neural representation for early SI to “work on,” which might in turn explain stronger additivity for this combination of conditions.

### Spatial context dominance

The aim of introducing the competitive conditions was to determine whether spatial or temporal factors exerted the stronger influence on perception. When both context types acted intraocularly (FS: intraocular, SI: intraocular same), the spatial context was the more relevant factor and strongly determined perception. Meanwhile, when both acted interocularly (FS: interocular and SI: interocular same), temporal context determined the percept more frequently. Of course, this seems to undermine the conclusion that spatial context was more important. Notably, this was the only condition where temporal context was deemed more influential and, as we argued before, the temporal factor FS was indeed not very weak in the interocular condition, as shown by the very small difference in the baseline between intra- and interocular FS. When the combination was FS intraocular and SI interocular same (see Figure 6, left panel), neither influence won over the other, despite the spatial context also being especially weak. We would therefore still argue that spatial context is the more dominant influence.

The question remains: why is spatial context more influential? One potential factor is that the spatial context always co-occurs with the bistable stimulus during the probe, and stimuli that appear together are grouped together (Wagemans et al., 2012). This is further supported by evidence from other bistable perception studies. Alais and Blake showed that grouping is enhanced when the changes in stimulus contrast are the same or correlated compared to uncorrelated (Alais & Blake, 1999).

Our strong spatial context effect also aligns with evidence from higher-level vision. He, Richter, Wang, and de Lange (2022) examined how spatial and temporal regularities modulate neural responses along the ventral visual stream. They made use of the phenomenon that the neural response to object stimuli that are predicted by the context and are thus expected, is smaller than that of surprising objects (that are not predicted by the context). They modulated the context type: it could be either spatial (neighboring objects) or temporal (objects following each other in time). They found that both forms of context independently suppressed activity for expected stimuli – consistent with predictive-coding models – but that the suppression was markedly stronger and more widespread for spatial regularities, also extending to object-selective cortices. Temporal expectations, in contrast, produced weaker and more localized effects (He et al., 2022). These findings suggest that the visual system might generally weight spatial information more heavily than temporal cues. It remains to be tested whether spatial dominance may reflect a broader principle that extends throughout the visual hierarchy.

### Clarity ratings: Enhancement through additivity and reduction through competition

We also investigated the influence of additive and competitive dynamics on the clarity ratings. Previously, we only compared additive with competitive trials and found higher clarity for additive trials (Beckmann et al., 2025b). Here, we compared the results to the baseline for a more nuanced conclusion. We found that competitive constellations reduced the clarity and that additive constellations increased the clarity compared to the baseline. This main effect could be found for all SI manipulations (surround contrast and ocularity). For the different FS ocularity measures, this was mostly true as well. However, the clarity seems to plateau in the intraocular condition in additive trials, which might be the result of very high clarity in the FS baseline. These findings further support the notion that temporal and spatial contextual influences contribute jointly to perceptual organization. Their effects are reflected not only in percept choice, but also in the subjective quality of the resulting perceptual experience.

### Limitations and future directions

Several limitations should be considered when interpreting the present findings. A primary motivation of this study was to identify a manipulation that could be applied analogously to both FS and SI. Ocularity fulfilled this role experimentally, allowing direct comparison of temporal and spatial context along a shared dimension. However, ocularity did not affect the two influences to the same extent. Whereas interocular FS remained comparatively robust, SI was substantially reduced under interocular presentation. Consequently, ocularity did not provide a means of equating the strengths of the two influences across paradigms. However, this asymmetry constituted one of the central findings of the present study, suggesting that eye-of-origin information contributes differently to temporal and spatial influences.

A second limitation concerns the scope of the paradigm. The present study focused on rivalry onset rather than sustained dynamics. This was the result of focusing the experiment around FS which is by nature restricted to the onset. Although onset rivalry and sustained rivalry share many characteristics, previous work (Carter & Cavanagh, 2007) suggests that the mechanism governing initial perceptual selection may differ in part from those underlying later perceptual alternations. The present conclusions should therefore be interpreted primarily as applying to context effect on onset rivalry. It should be noted, however, that in the previous publication, we replicated effects otherwise only shown during sustained rivalry also for the onset in regards to SI (Beckmann et al., 2025b).

Because we did not use trials with unambiguous perception as catch trials, we cannot experimentally exclude the possibility of a response bias (instead of responses based on perception). Notably, this approach is consistent with the relevant studies, which likewise did not include unambiguous catch trials (e.g., Brascamp et al., 2007; Ikeda et al., 2009; Ikeda & Morotomi, 2002). We therefore acknowledge response bias as a potential limitation of the present design. At the same time, we consider it unlikely that response bias (rather than the participants’ perception of the target) can account for the observed pattern of results in the 2AFC task. In particular, following up on their responses in the 2AFC task, participants made graded and heterogeneous use of the clarity rating scale, with clarity judgments varying systematically across experimental conditions rather than showing a random response pattern (as might be expected if the 2AFC response did not reflect a perceptual decision). Although these clarity ratings cannot completely rule out an influence of response bias in the preceding 2AFC responses, they provide converging evidence supporting our interpretation of the observed effects.

Furthermore, future work may benefit from using more graded manipulations of eye-of-origin weighting. For example, systematic interocular contrast imbalances may provide a means of continuously varying the contribution of each eye, allowing a more precise characterization of how temporal and spatial contextual influences depend on binocular interactions.

In this study, we used FS as an example for a temporal influence and SI as an example for a spatial influence. Future studies should demonstrate whether the combined effects found for FS and SI generalize to other forms of combined temporal and spatial influences. In the temporal domain, other influences worth exploring are perceptual memory (Pearson & Brascamp, 2008), previous adaptation (Long & Moran, 2007), higher-order influences (Chastain & Burnham, 1975), or the influence of previous stereopsis on rivalry dynamics (Jiang & Meng, 2025). In the spatial domain, one might think of perceptual coupling (Eby, Loomis, & Solomon, 1989; Grossmann & Dobbins, 2003; Pastukhov & Carbon, 2022), effects of neighboring unambiguous stimuli (Ouhnana & Kingdom, 2016), or parallel to the abovementioned temporal effect – the influence of spatially adjacent stereopsis cues (Jiang & Meng, 2023).

## Conclusions

In the present study, we individually examined the effects of various ocularity conditions in spatial and temporal context cues and their combined influence when presented to the same or different eyes. Although ocularity could be manipulated analogously in FS and SI, it affected the two paradigms differently. Whereas FS remained comparatively robust under interocular presentation, SI was substantially weakened, indicating a stronger dependence of spatial context on eye-of-origin information. When temporal and spatial context favored the same percept, their effects were largely additive and often produced highly predictable perceptual outcomes despite the physical ambiguity of the rivalry stimulus. In competitive situations, spatial context generally exerted the stronger influence, although the unexpectedly robust interocular FS effect dominated over the interocular SI effect. Finally, additive contextual influences increased perceptual clarity, whereas competing influences reduced it. Taken together, the present findings suggest that temporal and spatial contextual influences differ in their sensitivity to eye-of-origin information and support the view that spatial context plays a particularly important role in shaping perceptual selection during rivalry onset.

## Supplementary Material

Supplement 1
