# Supplementary material for: Ocularity modulates the dominance of spatial over temporal influences in binocular rivalry
Source: J Vis. 2026 Oct 9;26(10):9. doi: 10.1167/jov.26.10.9 (PMC13676251; doi:10.1167/jov.26.10.9)
Supplement: Supplementary file 1 [file jovi-26-10-9_s001.pdf]

Supplemental Material

S.1 Additional Information for the Main Experiment

Figure S1

Example trial of the (A) SI baseline and (B) FS baseline.

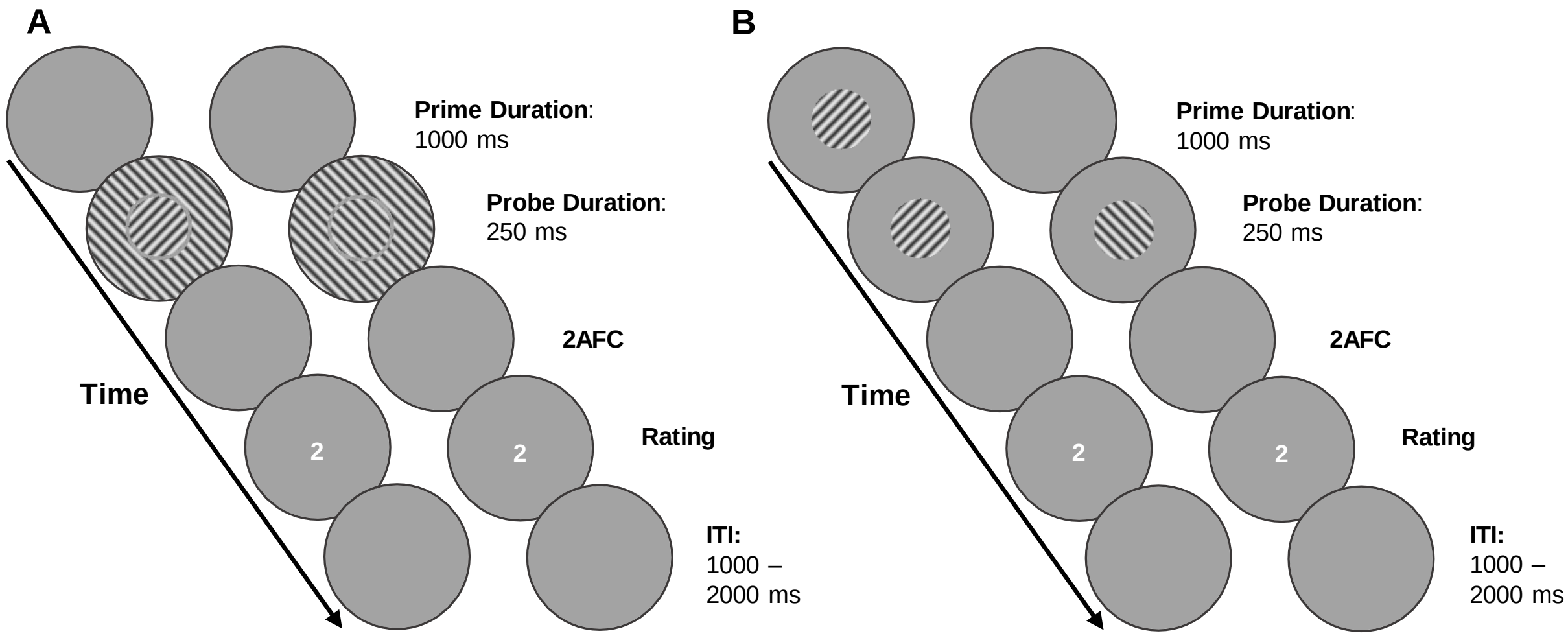

Note. This figure demonstrates two example baseline trials. The left circles represent what was presented to the left eye, and the right circles represents stimuli shown to the right eye, a circular fusion frame (not depicted here) surrounded these stimuli. (A) An empty grey patch was shown during the "prime" for 1000 ms. During the duration of the probe, participants saw two central gratings (rivalry target) that were rotated CW or CCW. In this condition, the central target was surrounded by a grating. This surround could also be only presented to one stimulus either iso-oriented or orthogonal (see Figure 1 in the publication). The probe was followed up with a 2AFC. Here, participants had to indicate the dominant percept during the probe. Afterward, participants recorded a perceptual clarity rating between 0 and 3. Each trial ended in an ITI randomly ranging between 1000 and 2000 ms. (B) Here, no surround was presented during the probe. Instead, during the prime one of the gratings presented at probe was shown. This could be intraocularly (as depicted here), i.e., within the same eye, or interocularly, i.e., the grating would switch the eye from prime to probe presentation.

S.2 Supplemental Experiment

S.2.1 Methods

Participants. In our previous publication (Beckmann et al., 2025b), we focused solely on intra-ocular presentation to avoid an overly lengthy manuscript, deferring the analysis of ocularity effects to the present work, where they can be supplemented by the data of the main experiment, in which ocularity was modulated in both FS and SI. Consequently, the participants of the supplemental experiment are the same as those in Exp. 2 in Beckmann et al. (2025b). In total, 17 participants (14 female and 3 male) took part in the experiment. Ages ranged from 20 to 33 years, averaging 23.06 years (SD = 3.94 years). Only right-handed participants who reported no history of psychological or neurological disorders were included in the study. All our participants had normal or corrected-to-normal visual acuity. Compensation was provided as 10€ per hour or course credit. The study was approved by the local ethics board of the Department of Psychology of the Ludwig-Maximilians-Universität München.

Apparatus. The apparatus was the same as in the experiment described in the main text.

Stimuli. The stimuli were the same with one notable exception. SI was not manipulated with its regard to ocularity but rather the contrast of the surround was vaired. The surround could be presented at either high or low contrast. In the low contrast surround condition, the surround was presented at 10% Michaelson contrast. A tiny gray gap spatially separated the central grating and the surround. This gap was introduced to ensure participants knew which part of the stimulus to report their perception. The stimuli were surrounded by a dark gray fusion frame with black circles to reinforce binocular fusion. This fusion frame increased the size of the complete stimulus to 3.7° va. The mean luminance of all gratings at their respective positions on the screen was 100 cd/m^2 at 90% (high) contrast and 101 cd/m^2 at 10% (low) contrast.

Procedure. The procedure was largely the same. However, after the prime stimulus, the probe was shown for 120, 250, 500, or 1000 ms. During pilot testing, participants were explicitly asked whether they experienced rivalry-like perceptual switches during the probe presentation. None of the participants reported such switches. We have two relevant conditions: (1) FS ocularity (intraocular / interocular presentation of prime and probe) and (2) surround contrast: contrast of the annulus (10%, 90%). The duration of probe presentation was also varied (120 ms, 250 ms, 500 ms, 1000 ms), but these results were already analyzed in our previous publication (Beckmann et al., 2025b). Since there was no interaction between probe duration and FS ocularity and no general probe duration effect, we focus solely on the FS ocularity manipulation, averaging over probe durations. Several control conditions were introduced to eliminate influences unrelated to the variables of interest: (3) the grating orientation of the prime stimulus (CW, CCW), (4) The eye viewing the prime stimulus (left, right), and (5) the grating type of the surrounding annulus (CW, CCW). Trials were equally distributed across all resulting condition combinations. The trial order was fully randomized. The experiment was split into 16

blocks of 52 trials each, resulting in 832 trials in total. Completing this took up to 60 minutes (including instructions) for each of the two experimental sessions.

S.2.2 Results

2.2.1 Baseline validation. Baseline analyses were performed to verify that the supplemental experiment reproduced the expected FS and SI effects as previously found by other research groups and ourselves (Beckmann et al., 2025a, 2025b; Ikeda et al., 2009; Wolfe, 1984) before examining their interaction. As in the main experiment, separate analyses were conducted for the FS and SI baseline conditions. For the FS baseline, the probe presented without a surround, allowing the flash suppression effect to be assessed in isolation. To investigate this, we fitted GLMMs with a binomial response (percept in line with FS [1] vs. not in line with FS [0]) and participant as a random intercept.

First, to show that the percept not shown during the prime dominated perception across ocularity conditions, we looked at the model with only a random intercept for "participant" without any fixed effects. Importantly, a Wald test on the estimated intercept indicated that participants were more likely to perceive the central grating not shown during the prime ($\beta = 2.11$, $SE = 0.19$, $z = 11.42$, $p < .001$, 95% CI [1.75, 2.47]); this grating was dominant in 87.35% of the trials. Thus, we could find the basic FS effect.

Next, we wanted to see if we could replicate the FS ocularity effect, i.e., whether intraocular prime and probe presentation lead to a stronger FS effect than interocular presentation (Ikeda et al., 2009). This was indeed the case for the observed data, see Figure S2A. To test this effect statistically, we fitted a GLMM with FS ocularity as a fixed effect and participant as a random factor – the reference was set to the hypothesized weakest condition (interocular). A likelihood ratio test revealed a significantly better fit for this model, compared to the random-intercept model, LRT: $\chi^2(1) = 60.90$, $p < .001$, $R^2_{McFadden} = 0.026$, $\Delta AIC = 58.90$. The estimated log-odds for the intraocular condition were significantly higher than for the interocular reference, $\beta = 0.87$, $SE = 0.11$, $Z = 7.58$, $p < .001$, Figure S2A. Thus, these results are consistent with previous findings on FS ocularity, namely a stronger effect of intraocular FS compared to interocular FS (Ikeda et al., 2009).

We have already calculated the baseline validation for SI in our previous publication (Beckmann et al., 2025b), but we will repeat the core finding here for convenience. According to SI, participants should see the nonmatching central stimulus in most trials, i.e., the stimulus orthogonal to the surround grating. This was confirmed by our data, see Figure S2B. We again fitted GLMMs with a binomial response (percept in line with SI [1] vs. not in line with SI [0]). This nonmatching boost effect (77.67% (observed data), 78.37% (tested model value) over all SI baseline trials, irrespective of contrast) could be found for the SI baseline condition (i.e., no prime), as indicated by a significant Wald test on the random-intercept GLMM, i.e., a test against chance, here 50% ($\beta = 1.29$, $SE = 0.12$, $z = 11.23$, $p < .001$, 95% CI [1.07, 1.52]).

Figure S2

Baseline condition results for FS (A) and SI (B).

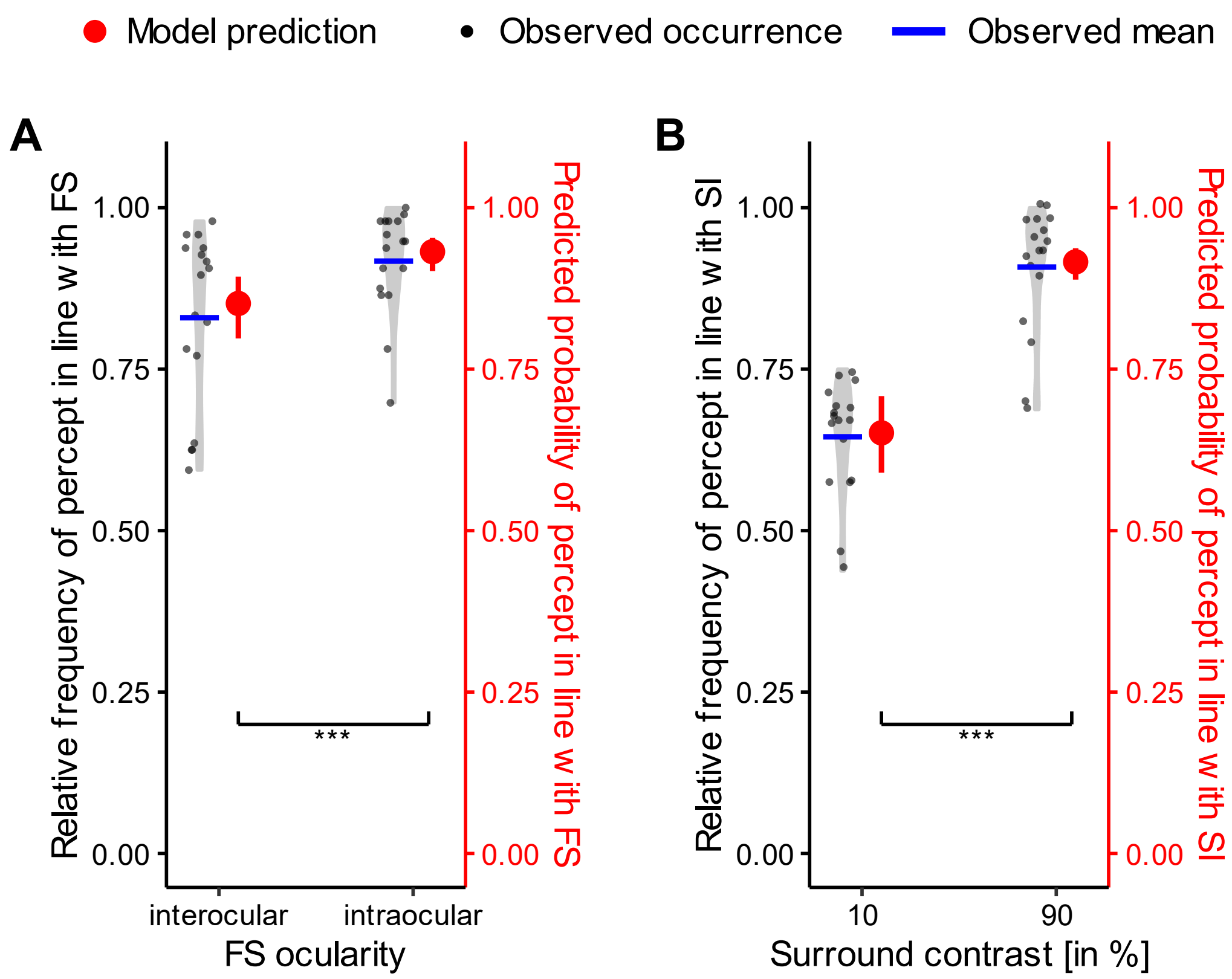

Note. (A) Relative frequency of the percept reported in line with the flash suppression (FS) paradigm (black left y-axis) and corresponding model predictions (red, right y-axis) across varying FS ocularity conditions (interocular and intraocular) for the FS baseline. Light-gray violin plots represent the distribution of individual data, with jittered black dots indicating the observed values per participant, and blue horizontal lines showing the observed condition means. Red points with error bars show model-predicted means and their 95% confidence intervals. Statistical significance is based on Bonferroni-corrected planned comparisons of model predictions; * = $p_{adj} < .05$, ** = $p_{adj} < .01$, *** = $p_{adj} < .001$. (B) Same as (A), but for the surround inhibition (SI) paradigm. Surround contrasts are ordered from weak (10%) to strong (90%) in terms of hypothesized SI strength. All visual conventions from panel (A) apply.

Furthermore, we fitted a model with the fixed effect of surround contrast in addition to the random effect for participant, again with the weakest effect (10% surround) as a reference. The LRT test revealed a significantly better fit for this model compared to the random-intercept model, $\chi^2(1) = 237.23$, $p < .001$, $R^2_{McFadden} = 0.105$, $\Delta AIC = 235.23$. The estimated log-odds for the 90% surround revealed a significantly stronger SI effect compared to the reference value (10% surround), $\beta = 1.77$, $SE = 0.13$, $z = 14.00$, $p < .001$, see Figure S2B. This shows that the high contrast surround yielded a stronger influence on perception than the low contrast surround.

2.2.2 Additivity between spatial and temporal context. When the two factors FS and SI influence perception in the same direction, we expect more percepts in line with FS and SI than in the baselines (only FS or only SI, respectively). We first analyzed the effect of spatial context

(surround) on flash suppression. To do this, we created a new factor called "*additivity*" to test for additive effects on the baseline. This factor had the following three levels: (1) only FS, meaning the FS baseline condition. (2) FS with weak spatial context, meaning the prediction of FS with the addition of a surround with 10% contrast – the surround would further reinforce the same stimulus to dominate perception as the FS paradigm. (3) FS with strong spatial context, meaning the prediction of FS with the addition of a surround with 90% contrast. The factor "*FS ocularity*" was also included. Here, ocularity relates to the FS paradigm alone with the following levels: intraocular (the prime stimulus is presented to the same eye as during the probe) and interocular (the prime stimulus is presented to the other eye compared to presentation during the probe).

To test for the additivity effect, we fitted several GLMMs with a binomial response (percept in line with FS vs. not in line with FS). The best-fitting model was the full model, including additivity and ocularity as fixed effects, as well as their interaction. The LRT comparing the full model to the model without interaction revealed a significant improvement in fit, $\chi^2(2) = 64.91$, $p < .001$, $\Delta R^2_{McFadden} = 0.014$, $\Delta AIC = 60.91$. Post-hoc comparisons showed that when the FS effect was weak (i.e., interocular), additivity could be found, FS baseline vs. FS baseline + 10% surround contrast: $\beta = 0.55$, $SE = 0.12$, $z = 4.55$, $p_{adj} < .001$; FS baseline vs. FS baseline + 90% surround contrast: $\beta = 1.94$, $SE = 0.19$, $z = 10.26$, $p_{adj} < .001$, see the left panel in Figure S3. However, when the FS effect was strong (i.e., intraocular), probabilities were already very high and no significant differences between different levels of the additivity factor could be found, see the right panel in Figure S3. To sum up, interocular FS and SI worked additively. Intraocular FS, however, was already so strong that added SI had no further influence on perception.

For the SI paradigm, we defined the factor "*additivity*" with the following three levels: (1) baseline without any prime (only SI), (2) the SI baseline plus interocular FS (SI + presumed weak FS), and (3) the SI baseline plus intraocular FS (SI + presumed strong FS), again calculated for each level of the surround contrast (factor "*contrast*"). If an additivity effect is present, participants should report the percept in line with both FS and SI more frequently in the additive conditions than in the SI baseline. Again, we fitted several GLMMs with a binomial response (percept in line with SI vs. not in line with SI) and the model including both fixed effects ("*additivity*", "*ocularity*") and their interaction term provided the best fit: LRT comparing the full model against the model without the interaction term: $\chi^2(2) = 82.82$, $p < .001$, $\Delta R^2_{McFadden} = 0.018$, $\Delta AIC = 78.82$. All but one of the post-hoc comparisons were significant, see Figure S4. This non-significant comparison occurred in the 90% contrast condition (right panel), between baseline and baseline + intraocular FS. Inspection of the individual participant data revealed that one participant showed a response pattern that deviated markedly from both the overall trend and their responses in the remaining conditions. As a robustness check, we repeated the analysis after excluding this participant. Although the comparison between baseline and baseline

+ intraocular FS reached statistical significance (z = 3.54, p < .001), we found no objective reason to exclude the participant and therefore retained all participants in the reported analyses. All in all, when the spatial context was weak (10% surround contrast), both inter- and intraocular FS significantly increased the likelihood of the predicted percept. When the spatial context was strong (90% surround contrast), only the interocular FS condition contributed to an additive effect.

Figure S3

Additivity of spatial context (SI) on temporal context (FS).

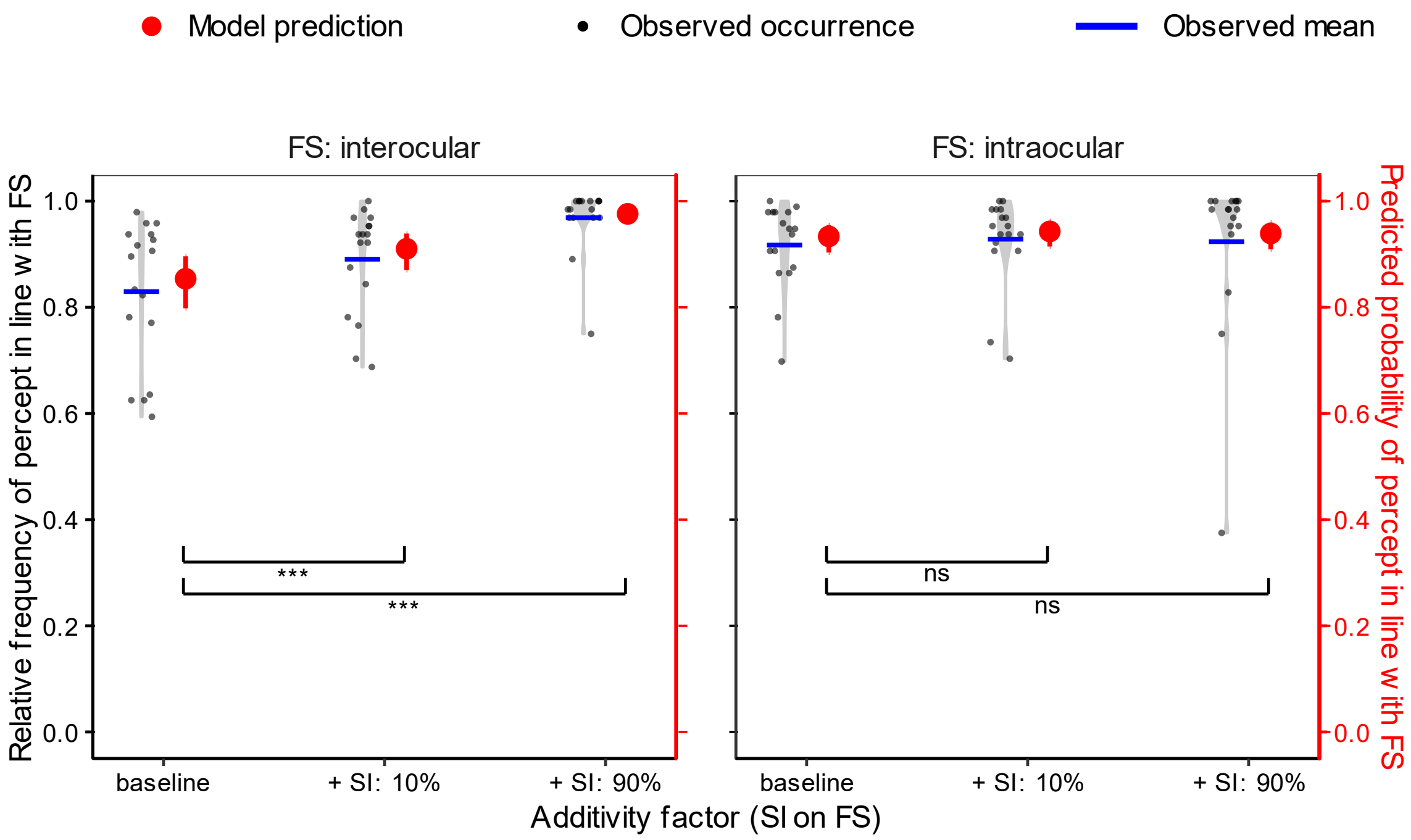

Note. Relative frequency of the percept reported in line with the flash suppression (FS) paradigm (black left y-axis) and corresponding model predictions (red, right y-axis) across different levels of the additivity factor. The x-axis is ordered from weakest to strongest, starting with the pure FS baseline to FS-baseline with the weaker SI condition (10%) and then with the strongest SI condition (90%). Light-gray violin plots represent the distribution of individual data, with jittered black dots indicating the observed values per participant, and blue horizontal lines showing the observed condition means. Red points with error bars show model-predicted means and their 95% confidence intervals. Statistical significance is based on Bonferroni-corrected planned comparisons of model predictions; * = $p_{adj} < .05$, ** = $p_{adj} < .01$, *** = $p_{adj} < .001$.

Of note, when the high contrast surround and the interocular condition were combined, perception became almost completely predictable for almost all observers, i.e., all but two observers saw the predicted percept in almost all trials (96.90% mean observed occurrence, 97.49% model prediction). This shows that using additive influencing factors could be a good method of disambiguating a stimulus at a perceptual level while retaining its (theoretically) ambiguous nature at the retinal level. Also, surprisingly, in combination with the 90% surround, the interocular condition was stronger than the intraocular condition (z = -4.60, p < .001).

Figure S4

Additivity of temporal context (FS) on spatial context (SI).

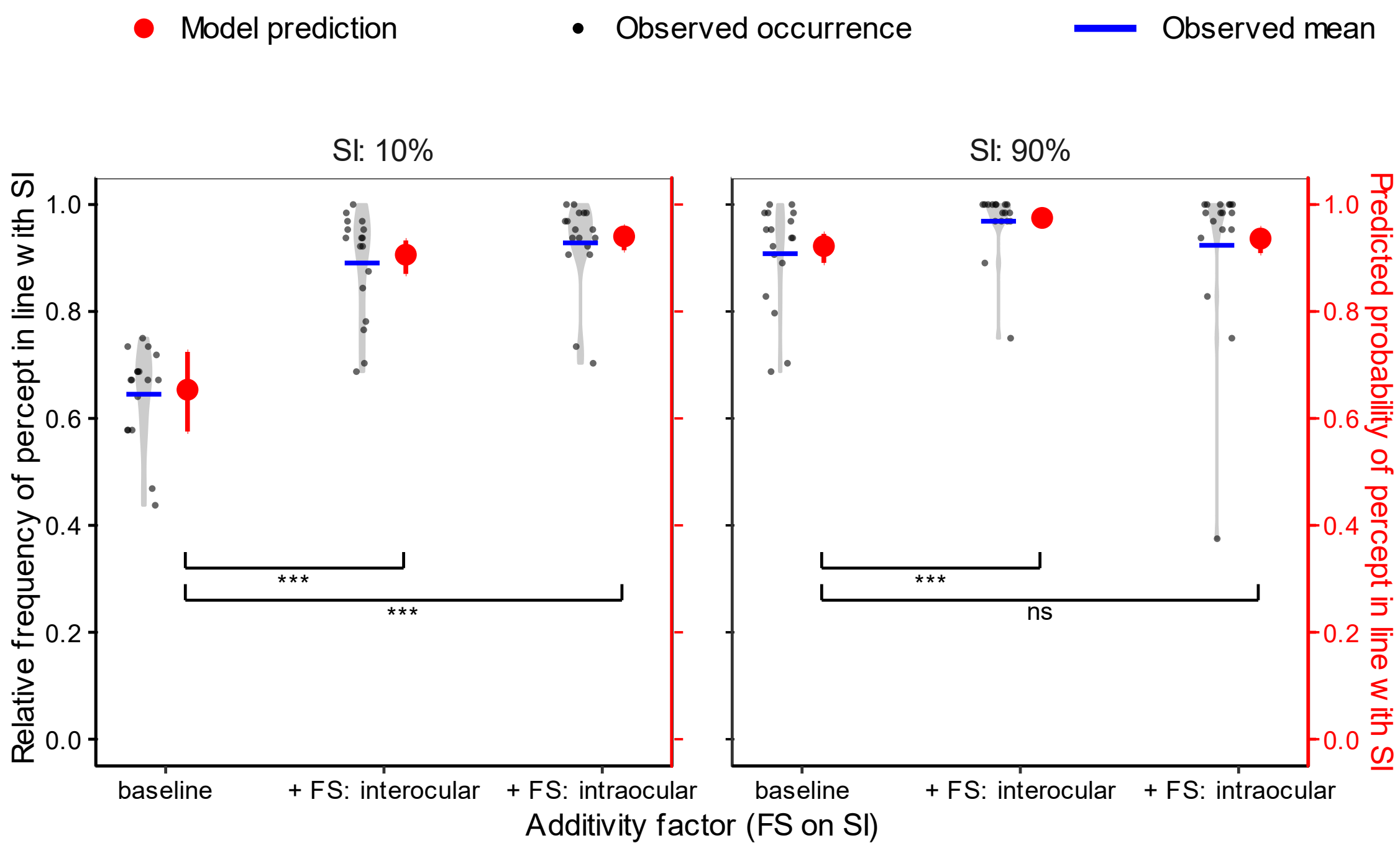

Note. Relative frequency of the percept reported in line with the surround inhibition (SI) paradigm (black left y-axis) and corresponding model predictions (red, right y-axis) across different levels of the additivity factor. The x-axis is ordered from presumed weakest to strongest, starting with the pure SI baseline, to the SI-baseline with the interocular FS condition, to the SI-baseline with the intraocular FS condition. Light-gray violin plots represent the distribution of individual data, with jittered black dots indicating the observed values per participant, and blue horizontal lines showing the observed condition means. Red points with error bars show model-predicted means and their 95% confidence intervals. Statistical significance is based on Bonferroni-corrected planned comparisons of model predictions; * = $p_{adj} < .05$, ** = $p_{adj} < .01$, *** = $p_{adj} < .001$.

2.2.3 Competition between spatial and temporal contexts – SI yields stronger influence than FS. Complementary to additive trials, there were also trials in which FS and SI influenced perception in different directions. For example, trials with a CCW-rotated surrounding grating (SI) and a CW-rotated prime grating (FS). Spatial context would boost the CW probe grating to dominate perception, while temporal context would favor the CCW grating. Investigating the actual percepts provides insight into the type of context the brain prioritizes.

To investigate any preferences, we fitted a series of GLMMs predicting whether the participants' responses followed the SI-consistent percept. Please note that we might as well have chosen FS-consistent percepts, since all decisions that are not in line with SI are automatically in line with FS; statistically, it makes no difference and only affects the sign of the log-odds. Since this is the case, we adapted the y-axis in Figure S5: values above the 0.5 show percepts predicted by SI, below the line percepts predicted by FS. Thus, one can directly see which effect dominated perception in which combination of conditions.

Both surround contrast and FS ocularity contributed significantly to the model fit. Adding FS ocularity to a spatial only model significantly improved the fit (LRT: $\chi^2(1) = 7.97$, $p = .005$, $\Delta R^2_{McFadden} = 0.001$, $\Delta AIC = 5.97$) and so did adding surround contrast to the FS only model (LRT: $\chi^2(1) = 1068.30$, $p < .001$, $\Delta R^2_{McFadden} = 0.181$, $\Delta AIC = 1066.25$). As indicated by the vastly increased $\Delta R^2_{McFadden}$, the contribution of spatial context to the temporal context was much stronger than that of the temporal context to the spatial context. This can be attributed to the larger difference in modulation strength between conditions in the spatial case than in the temporal case. Adding the interaction term significantly increased the fit further, LRT: $\chi^2(1) = 4.17$, $p = .041$, $\Delta R^2_{McFadden} = 0.001$, $\Delta AIC = 2.17$. Looking at Figure S6, it might be due to a slightly stronger difference between intra- and interocular FS in the 10% than in the 90% contrast condition. The best fitting model was thus the full model, including fixed effect of surround contrast (spatial context, SI), FS ocularity (temporal context, FS), and their interaction, along with the random intercept for the participant.

We used multiple levels of spatial and temporal influence to allow for a more nuanced comparison of their relative strengths. Relying on a single strength for each type of influence could have led to misleading conclusions, as any observed difference might simply reflect the arbitrary choice of strength rather than a true underlying effect. To more accurately assess the relative impact of spatial versus temporal influences, we searched for conditions that were equally strong in the baseline (see Figure S2) to then see which influenced perception more strongly under competitive conditions. To this aim, we conducted paired Bayesian t-tests to compare all combinations of SI baseline and FS baseline trials. There was only one pair of baseline conditions where the influences could be considered approximately equal, i.e., they influenced perception equally strongly (Bayes factor in favor of the null hypothesis, $BF_{01} > 3$; Jeffreys (1998)): intraocular FS (91.70%, see Figure S2, panel A) and 90% surround contrast (90.80%, see Figure S2, panel B), $BF_{01} = 3.65$. This can also be seen in Figure S5, rightmost panel: the light and dark green lines are approximately equidistant from the 0.5 line. All other combinations had a BF_{01} below 0.55.

This, however, needs to be interpreted with caution: the matching baselines might not reflect similar strengths, but – as they are both quite high – a ceiling effect in one or both conditions. Looking at this specific condition combination within the competitive trials (Figure S5, rightmost panel) revealed a bias in favor of spatial context, consistent with the results from our previous publication (Beckmann et al., 2025b): most percepts were in line with SI (i.e., data above the 0.5 line). This was also statistically confirmed with a Bayesian paired t-test on the competitive data, revealing strong evidence (Jeffreys, 1998) in favor of a larger effect of the spatial compared to the 0.5 chance level: $BF_{10} = 44.82$. The difference from the SI baseline (light green dashed line) was also much smaller than from the FS baseline (dark green dashed line). As argued above, we cannot rule out the possibility that this was in part due to a ceiling

effect in the SI baseline masking an even stronger SI effect, but it provides tentative evidence of spatial superiority.

Figure S5

Results from competitive trials.

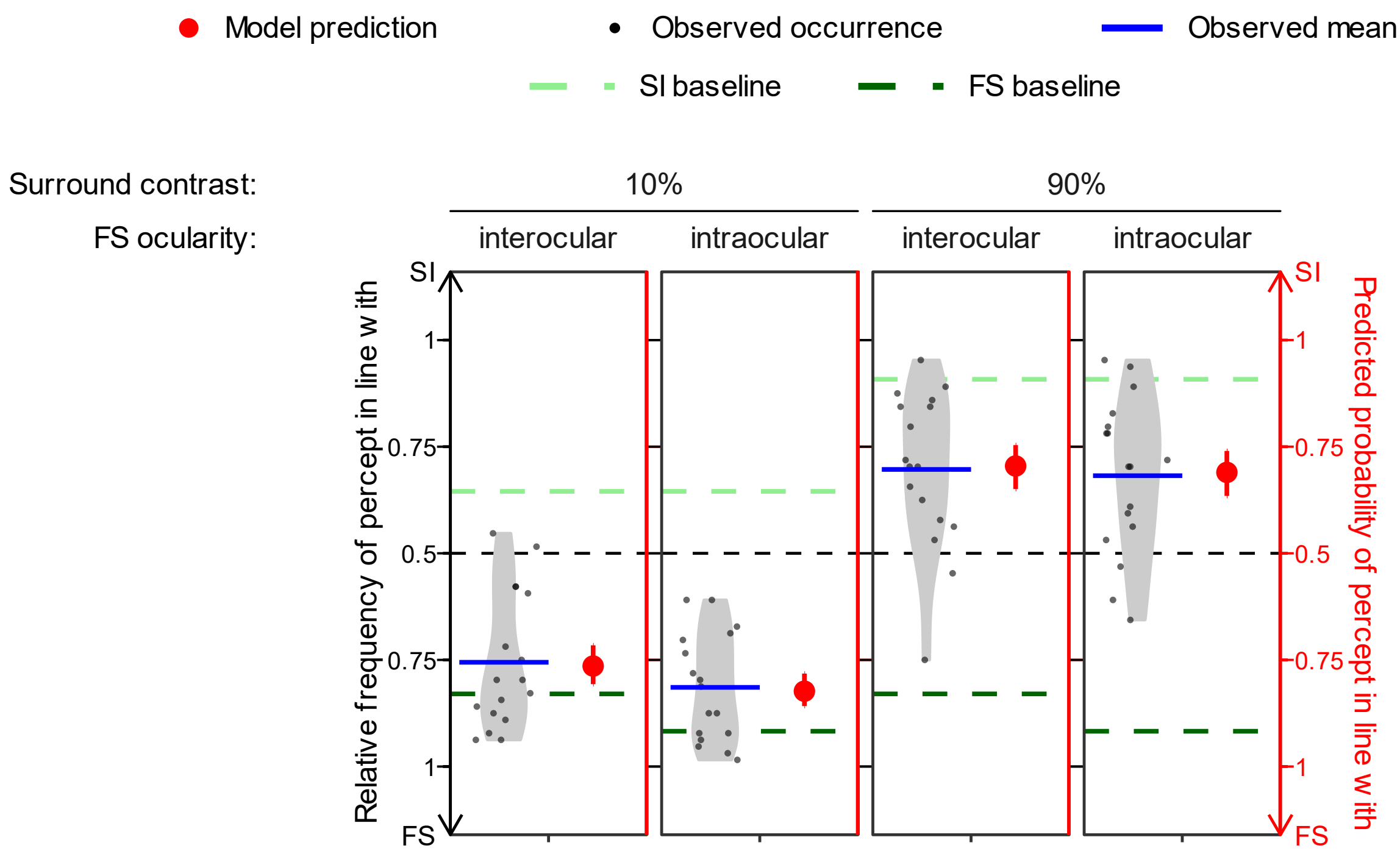

Note. Relative frequency of the percept (black left y-axis) per participant and corresponding model predictions (red, right y-axis) across different levels of the flash suppression ocularity and surround contrast. Light-gray violin plots represent the distribution of individual data, jittered black dots indicate the observed values per participant, and blue horizontal lines show the observed condition means. Red points with error bars show model-predicted means and their 95% confidence intervals. 0.5 on the y-axis means that SI and FS had the same influence on perception (visualized by a black dashed line). Values above this line indicate perceptual effects consistent with SI dominance, while values below this line reflect FS dominance. For reference, the average SI baseline values were plotted as light green dashed lines. This illustrates that adding the competitive factor FS reduced perception in line with SI substantially compared to said SI baseline. This effect was stronger for the 10% than for the 90% surround contrast; in fact, so much stronger that in the 10% contrast condition, percepts were mostly in line with FS. The average FS baseline values were plotted as dark green dashed lines. The 10% contrast surround had very little effect in “moving the data away from that line”; the 90% contrast surround, however, moved them across the 0.5 line, and most percepts were in line with SI. The differences between the two FS conditions were minimal.

2.2.4 Clarity ratings: Additive trials push, competitive trials reduce. Previously, we demonstrated how additive and competitive conditions affect the dominant percept. Next, we investigated how these dynamics influence the clarity of these percepts. To achieve this, we conducted two analyses. In the first, we compared clarity ratings in competitive trials, the SI baseline, and additive trials, and examined how these ratings were affected by surround contrast. To capture the clarity of the relevant percept, we used the following transformation: when the first response (which grating was dominant?) was in line with SI, the rating remained the same.

If the response was in line with FS, we applied sign reversal by multiplying it by -1 to obtain a negative value. This transformation is particularly important as clarity in competitive trials could reflect the clarity of the FS percept, particularly in weak SI conditions.

We fitted a series of cumulative link mixed models (CLMMs) to predict the transformed clarity rating, with a random intercept for participants. Potential fixed effects were surround contrast and trial type (additive trials, SI baseline trials, competitive trials). We began by including the fixed effects separately. Trial type alone significantly improved the model fit over the random incept-only model, LRT: $\chi^2(2) = 2872.08$, $p < .001$, $R^2_{McFadden} = 0.075$, $\Delta AIC = 2686.08$. This indicates that trial type had an effect on the clarity response. As can be seen in Figure S6A, this was due to additivity making percepts in line with SI more likely and clearer ($\beta = -1.03$, $SE = 0.05$, $z = -21.55$, $p_{adj} < .001$) and competition making them less likely and less clear ($\beta = -1.20$, $SE = 0.05$, $z = -24.94$, $p_{adj} < .001$). Similarly, including surround contrast in the model improved the model fit over the random intercept model, LRT: $\chi^2(1) = 1246.73$, $p < .001$, $R^2_{McFadden} = 0.032$, $\Delta AIC = 1244.73$. Figure S6A shows that this was due to clearer percepts in the 90% contrast condition. This effect was, however, smaller than the effect of trial type ($\Delta R^2_{McFadden} = 0.042$).

The best model was the full model, which included the interaction of contrast and trial type (LRT comparison of the full model with the model including only the two fixed effects: $\chi^2(2) = 230.17$, $p < .001$, $\Delta R^2_{McFadden} = 0.006$, $\Delta AIC = 226.17$). The small $\Delta R^2_{McFadden}$ shows that the interaction's contribution was minimal. It might be due to a smaller difference between competitive trials and the SI baseline in 10% surround contrast trials than in 90% surround contrast trials, see Figure S6A.

In the second analysis, we compared clarity ratings in additive trials, the FS baseline, and competitive trials, and examined how the ratings were affected by FS ocularity. Including trial type in the model significantly improved the model fit over a random-intercept model, LRT: $\chi^2(2) = 2766.99$, $p < .001$, $R^2_{McFadden} = 0.069$, $\Delta AIC = 2762.99$. Similarly to the first clarity analysis, the effect of trial type was large and driven by both a reduced clarity in competitive trials ($\beta = -1.40$, $SE = 0.04$, $z = -32.36$, $p_{adj} < .001$) and an increased clarity in the additive trials ($\beta = -0.75$, $SE = 0.04$, $z = -17.70$, $p_{adj} < .001$), see Figure S6B. Adding FS ocularity as a fixed effect to the model also significantly improved the model fit compared to the intercept model, LRT: $\chi^2(1) = 8.78$, $p = .003$, $R^2_{McFadden} = 0.000$, $\Delta AIC = 6.78$, with the interocular having lower ratings than the intraocular FS ocularity condition. The contribution of FS ocularity was minimal, compared to that of trial type ($\Delta R^2_{McFadden} = 0.068$, $\Delta AIC = 2756.20$).

Again, the CLMM, which included both fixed effects (trial type, FS ocularity) and their interaction provided the best fit: LRT comparison between the full model and the model including only the two fixed effects: $\chi^2(2) = 90.52$, $p < .001$, $\Delta R^2_{McFadden} = 0.002$, $\Delta AIC = 86.52$. And again, the influence of the interaction was minimal. Figure S6B suggests that it might be due

to a greater additive-FS baseline difference in inter- than intraocular trials. To summarize, percepts were consistently clearer in additive trials compared to the baseline conditions, which in turn had a higher clarity than competitive trials.

Figure S6

CLMM results for clarity rating in the supplemental experiment (A) with SI baseline and (B) FS baseline.

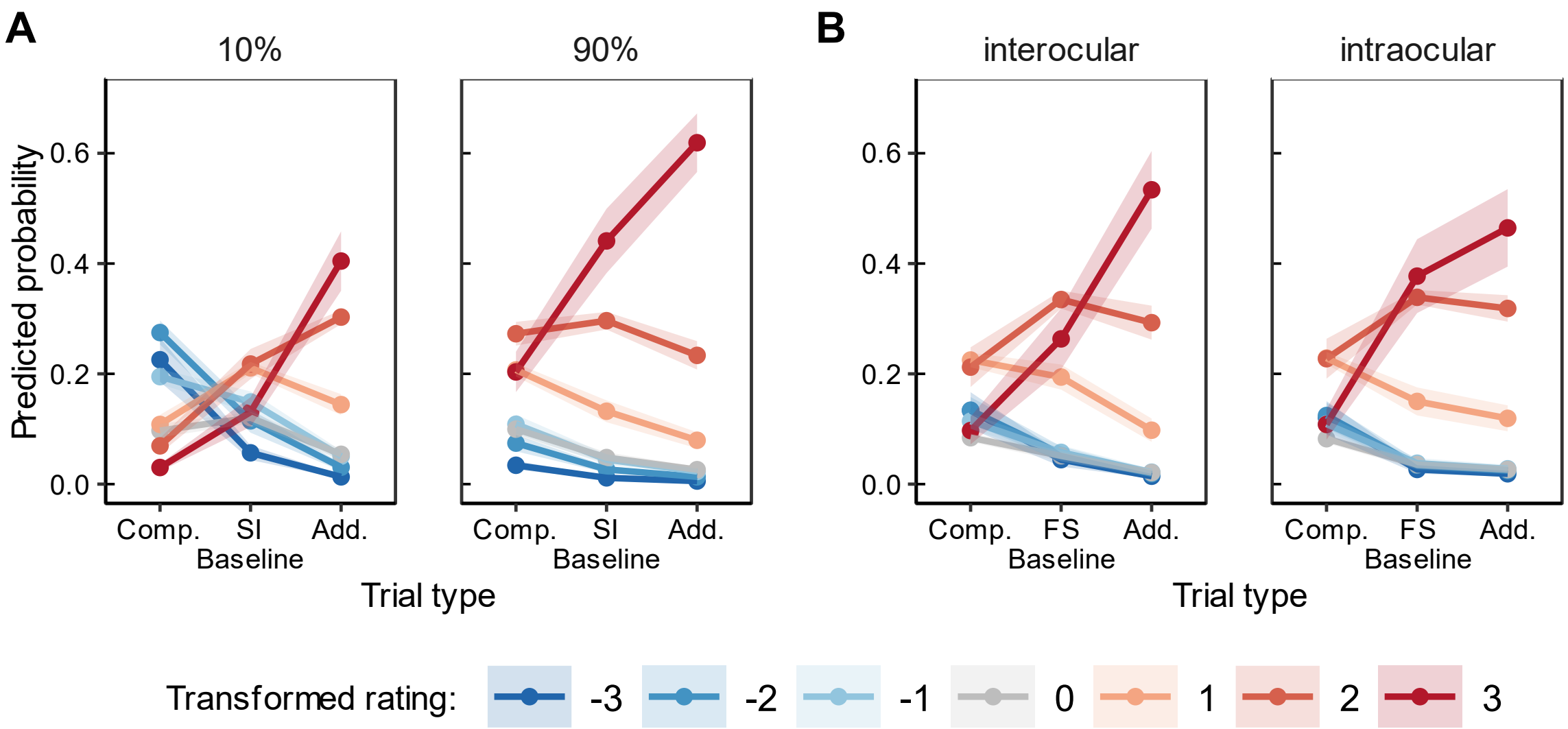

Note. Predicted probabilities of the transformed clarity ratings (-3 to 3) from a Cumulative Link Mixed Model (CLMM), as a function of trial type (x-axis) at different (A) surround contrasts and (B) FS ocularity conditions. Positive ratings (warm colored lines) represent ratings after responses in line with the respective influence (A: SI, B: FS), negative ratings (cold colored lines) represent ratings after responses not in line with this respective influence, the higher the absolute value of this rating, the higher the expressed clarity. Dots represent predicted probabilities for each rating level, with shaded ribbons indicating 95% confidence intervals (lines between the dots were only introduced for clearer presentation). Higher values on the y-axis indicate greater model-predicted likelihood of a given clarity rating. The equivalent plot showing the actual data (instead of model estimates) can be found in the supplemental material S.3 - Figure S8.

S.2.3 Discussion

The present study provides a nuanced replication and extension of prior findings on FS and SI. For FS specifically, our results align with earlier studies (Brascamp et al., 2007; Ikeda et al., 2009), demonstrating a stronger suppression effect for the intraocular condition compared to the interocular condition. This was most prominently shown in the baseline. The FS effect was significantly stronger in the intra- than in the interocular condition; however, the numerical difference was relatively small. Results from additive trials underline the surprising strength of the interocular FS condition: When temporal context was added to the already strong SI, only the interocular condition had an added effect (see Figure S4, right panel). The competitive condition confirmed that while intraocular FS was stronger, interocular FS remained

surprisingly strong. The significant interaction between FS ocularity and SI contrast indicated that, at low contrast, SI-consistent responses were higher for the interocular condition than for the intraocular condition, showing that SI-consistent responses were more strongly weakened by intraocular FS. At high contrast, the FS ocularity effect effectively disappeared with both conditions showing similarly high SI-consistent responses. When comparing the conditions to their baseline, the reduction through spatial context was stronger for the intraocular than for the interocular FS condition. To conclude, considering the FS baseline results, we could replicate a finding from the FS literature, namely that intraocular FS (stimulus-/pattern-based + eye-based effects) is stronger than interocular FS (only stimulus-/pattern-based effects) (Brascamp et al., 2007). Other studies have not consistently found this difference (Ikeda & Morotomi, 2002). In fact, in our study, the differences in the baseline were numerically quite small, suggesting that stimulus/pattern-based effects are far more important than eye-based effects.

Regarding SI, we replicated the well-documented surround contrast effect (e.g., Beckmann et al., 2025a, 2025b; Paffen et al., 2006), i.e., the bias towards perceiving the non-matching stimulus was drastically reduced for low compared to high contrast surrounds.

Concerning the combination of temporal and spatial factors, we successfully replicated several key findings from our earlier work (Beckmann et al., 2025b) and could extend the findings to a different modulation of the strength of FS (namely by modulating ocularity instead of the blank/probe duration):

Firstly, the general additivity of spatial and temporal context effects was further confirmed. At least in the weaker conditions, respectively, the added second factor (FS) led to increased influence on perception. Additionally, for the high contrast condition, the comparison between the SI baseline and the SI baseline with an interocularly presented prime demonstrated additivity. Therefore, while additivity is most prominent in weak conditions, it is not restricted to them.

Secondly, in competitive trials, spatial context was again shown to be the stronger influencing factor. When spatial and temporal context cues were similarly strong in isolation (based on the relative frequencies in the respective baseline conditions), spatial context cues were more influential in competition. In the case of low contrast surrounds, however, FS mostly determined perception (in both FS conditions, intra- and interocular).

Thirdly, clarity ratings were higher in additive trials than in competitive trials. We could further demonstrate in this study that this was driven by both a reduction in clarity in the competitive trials and an enhancement in the additive trials (compared to baseline trials, respectively).

These replications strengthen confidence in the reliability of our previous results (Beckmann et al., 2025b), e.g., the greater importance of spatial context cues compared to

tem-poral cues, as demonstrated by results from competitive trials here and in (Beckmann et al., 2025b).

S.3 Additional clarity ratings data

Figure S7

Transformed ratings for clarity ratings in the main experiment.

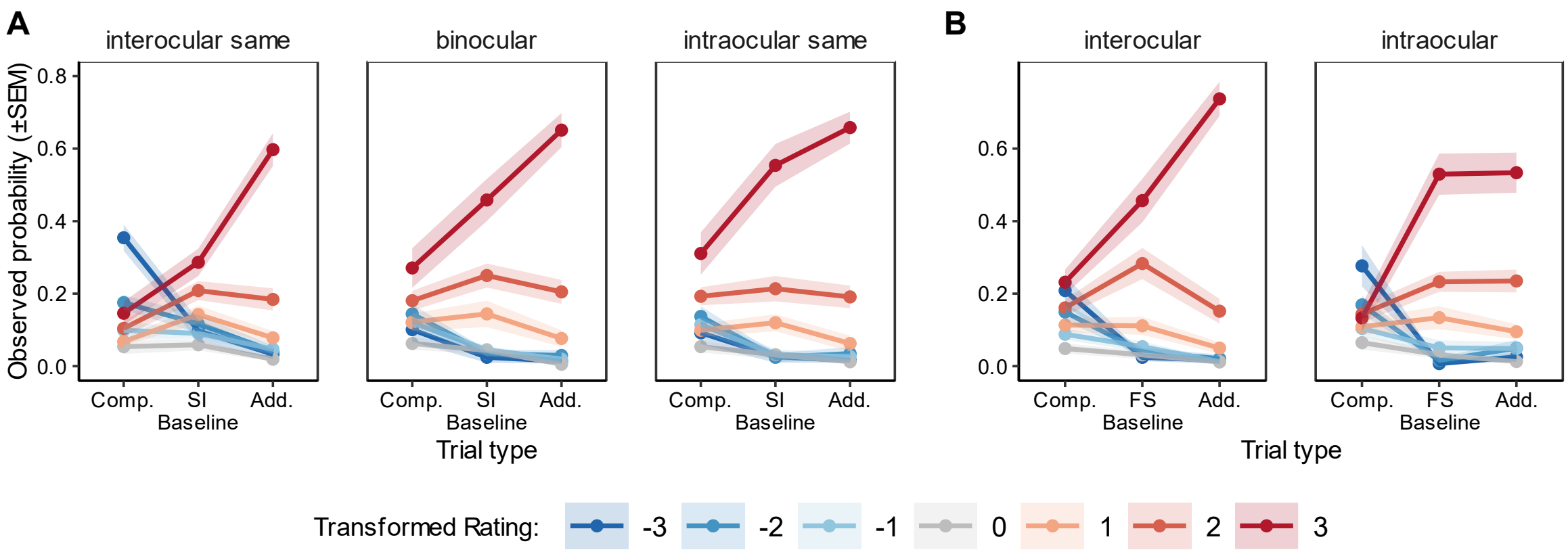

Note. Transformed clarity ratings (-3 to 3), as a function of trial type (x-axis), (A) SI ocularity, and (B) FS ocularity. Positive ratings (warm colored lines) represent ratings after responses in line with the respective influence (A: SI, B: FS), negative ratings (cold colored lines) represent ratings after responses not in line with this respective influence, the higher the absolute value of this rating, the higher the expressed clarity. Dots represent average occurrence for each rating level, with shaded ribbons indicating the standard error of the mean (SEM) (lines between the dots were only introduced for clearer presentation).

Figure S8

Transformed ratings for clarity ratings in the supplemental experiment.

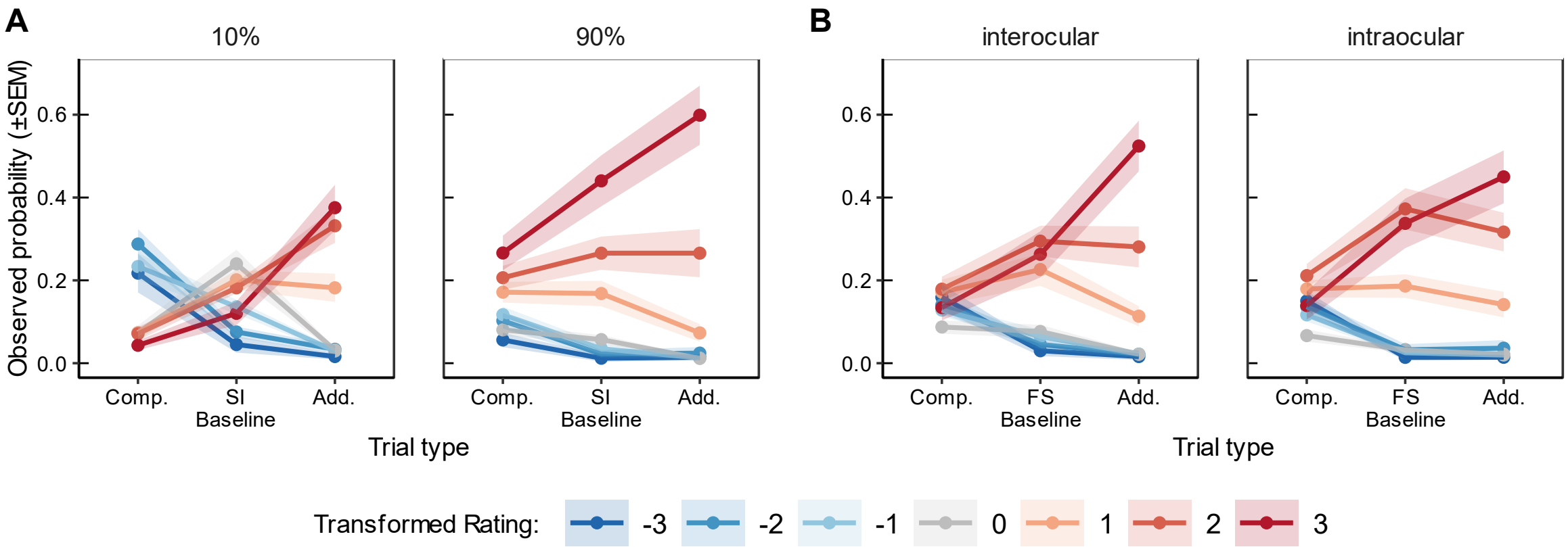

Note. Transformed clarity ratings (-3 to 3), as a function of trial type (x-axis), (A) surround contrast, and (B) FS ocularity. Positive ratings (warm colored lines) represent ratings after responses in line with the respective influence (A: SI, B: FS), negative ratings (cold colored lines) represent ratings after responses not in line with this respective influence, the higher the absolute value of this rating, the higher the expressed clarity. Dots represent average occurrence for each rating level, with shaded ribbons indicating the standard error of the mean (SEM) (lines between the dots were only introduced for clearer presentation).

References

Beckmann, L., Schenk, T., & Ludwig, K. (2025a). Measuring the influence of contrast, ambiguity, and side of spatial context on perceptual dominance during binocular rivalry. *Journal of Vision*, *25*(3), 6. https://doi.org/10.1167/jov.25.3.6

Beckmann, L., Schenk, T., & Ludwig, K. (2025b). When contexts collide: Spatial context prevails over temporal context in binocular rivalry. *Journal of Vision*, *25*(11), 11. https://doi.org/10.1167/jov.25.11.11

Brascamp, J. W., Knapen, T. H. J., Kanai, R., van Ee, R., & van den Berg, A. V. (2007). Flash suppression and flash facilitation in binocular rivalry. *Journal of Vision*, *7*(12), 1–12. https://doi.org/10.1167/7.12.12

Ikeda, K., & Morotomi, T. (2002). Color-specific filtering of rival binocular inputs induced by priming. *Japanese Psychological Research*, *44*(2), 57–65. https://doi.org/10.1111/1468-5884.00007

Ikeda, K., Ogata, Y., & Morotomi, T. (2009). Perceptual experience and temporal two stages in flash suppression for contour rivalry. *Psychologia*, *52*(1), 25–40. https://doi.org/10.2117/psysoc.2009.25

Paffen, C. L. E., Tadin, D., te Pas, S. F., Blake, R., & Verstraten, F. A. J. (2006). Adaptive center-surround interactions in human vision revealed during binocular rivalry. *Vision Research*, *46*(5), 599–604. https://doi.org/10.1016/j.visres.2005.05.013

Wolfe, J. M. (1984). Reversing ocular dominance and suppression in a single flash. *Vision Research*, *24*(5), 471–478. https://doi.org/10.1016/0042-6989(84)90044-0
